# Single-Cell RNA Sequencing of Peripheral Blood Mononuclear Cells From Pediatric Coeliac Disease Patients Suggests Potential Pre-Seroconversion Markers

**DOI:** 10.3389/fimmu.2022.843086

**Published:** 2022-03-15

**Authors:** Aarón D. Ramírez-Sánchez, Xiaojing Chu, Rutger Modderman, Yvonne Kooy-Winkelaar, Sibylle Koletzko, Ilma R. Korponay-Szabó, Riccardo Troncone, Cisca Wijmenga, Luisa Mearin, Sebo Withoff, Iris H. Jonkers, Yang Li

**Affiliations:** ^1^ Department of Genetics, University Medical Center Groningen, University of Groningen, Groningen, Netherlands; ^2^ Department of Computational Biology for Individualised Medicine, Centre for Individualised Infection Medicine (CiiM) & TWINCORE, Joint Ventures Between the Helmholtz-Centre for Infection Research (HZI) and the Hannover Medical School (MHH), Hannover, Germany; ^3^ Department of Immunohematology and Blood Transfusion, Leiden University Medical Center, Leiden, Netherlands; ^4^ Department of Pediatrics, Dr. von Hauner Children’s Hospital, Ludwig-Maximilians-Universität München (LMU) Klinikum Munich, Munich, Germany; ^5^ Department of Pediatric Gastroenterology and Nutrition, School of Medicine Collegium Medicum University of Warmia and Mazury, Olsztyn, Poland; ^6^ Coeliac Disease Center, Heim Pál National Paediatric Institute, Budapest, Hungary; ^7^ Department of Paediatrics, Faculty of Medicine and Clinical Center, University of Debrecen, Debrecen, Hungary; ^8^ Department of Medical Translational Sciences and European Laboratory for the Investigation of Food Induced Diseases, University Federico II, Naples, Italy; ^9^ Department of Internal Medicine and Radboud Institute for Molecular Life Sciences, Radboud University Medical Center, Nijmegen, Netherlands

**Keywords:** celiac disease, scRNAseq, PBMC, differential gene expression, pre-diagnostic biomarkers

## Abstract

Celiac Disease (CeD) is a complex immune disorder involving villous atrophy in the small intestine that is triggered by gluten intake. Current CeD diagnosis is based on late-stage pathophysiological parameters such as detection of specific antibodies in blood and histochemical detection of villus atrophy and lymphocyte infiltration in intestinal biopsies. To date, no early onset biomarkers are available that would help prevent widespread villous atrophy and severe symptoms and co-morbidities. To search for novel CeD biomarkers, we used single-cell RNA sequencing (scRNAseq) to investigate PBMC samples from 11 children before and after seroconversion for CeD and 10 control individuals matched for age, sex and HLA-genotype. We generated scRNAseq profiles of 9559 cells and identified the expected major cellular lineages. Cell proportions remained stable across the different timepoints and health conditions, but we observed differences in gene expression profiles in specific cell types when comparing patient samples before and after disease development and comparing patients with controls. Based on the time when transcripts were differentially expressed, we could classify the deregulated genes as biomarkers for active CeD or as potential pre-diagnostic markers. Pathway analysis showed that active CeD biomarkers display a transcriptional profile associated with antigen activation in CD4+ T cells, whereas NK cells express a subset of biomarker genes even before CeD diagnosis. Intersection of biomarker genes with CeD-associated genetic risk loci pinpointed genetic factors that might play a role in CeD onset. Investigation of potential cellular interaction pathways of PBMC cell subpopulations highlighted the importance of TNF pathways in CeD. Altogether, our results pinpoint genes and pathways that are altered prior to and during CeD onset, thereby identifying novel potential biomarkers for CeD diagnosis in blood.

## Introduction

Celiac disease (CeD) is a complex immune disorder triggered by gluten intake. It is characterized by inflammation and atrophy of the small intestine that severely impacts the quality of life of patients (1, 2). Even though CeD has a worldwide incidence of 1–1.5% (3), the only available treatment is a strict, life-long gluten-free diet (2).

Early diagnosis of CeD is key to minimizing its impact on patient quality of life because the persistence of intestinal damage is associated with complications such as bone abnormalities and malignancies (4) and CeD may hinder growth and development in children (5). After CeD diagnosis, patients follow a gluten-free diet, which is enough to stop villus atrophy and alleviate symptoms in most cases (2, 6). However, full recovery of the small intestine is only achieved in 50% of patients after one year of a gluten-free diet (6). Thus, diagnosis at an earlier stage of the disease, prior to the development of major villous atrophy, may decrease the burden on patients.

To date, diagnosis of CeD in adults mainly relies on serological markers found in blood, such as antibodies against tissue transglutaminase 2 (anti-TG2) (7), and on histopathological assessment of small intestinal villus atrophy and lymphocyte infiltration, both indicative of mucosal damage (8). However, CeD diagnosis is challenging in patients with IgA-deficiency, which is observed in about 2-3% of all CeD patients (9), and in patients with other (auto)immune-related diseases (7, 10, 11). Because of this limitation, novel biomarkers that can be detected at early stages of CeD-onset could improve the quality of life of patients, especially children, because villous atrophy and its consequences could be prevented. Ideally, such biomarkers would be present in tissues that can be collected in a minimally invasive manner, for instance blood.

Protein and RNA molecules are the most-studied blood biomarkers because these molecules can now be rapidly, accurately and affordably measured in virtually any type of sample (12). One advantage of RNA over proteins is that the limit of detection for RNA molecules is lower than that for proteins, which allows to use small quantities of input material, while sequencing-based technology for RNAs provides a genome-wide measurement. Additionally, there is already evidence that some RNA markers are predictive for CeD prior to villous atrophy (13) and could thus help individuals at high-risk of CeD to start the gluten-free diet before intestinal damage occurs and before the onset of major symptoms.

There are two types of RNA markers in blood: circulating ‘cell-free’ RNA (e.g. miRNAs or other RNA species that might be present in vesicles) and cellular/PBMC RNA (14). However, most studies that try to identify cellular RNA markers in blood rely on the analysis of mixed PBMC populations. The drawback of this approach is that transcriptional changes observed in bulk RNA are heavily impacted by changes in cell population frequencies, and transcriptomic changes in the minor populations may ‘snow under’ the transcripts measured in the more abundant cell types (15). Single-cell RNA sequencing (scRNAseq) makes it possible to study complex tissues like blood at cellular resolution, which allows the unbiased identification of cell type–specific transcriptomes in their tissue (blood) context (16), e.g. in a comparison of disease cases versus controls at different timepoints.

Here, we studied dynamic transcriptional changes in single cells of pediatric individuals at increased risk of developing CeD both before and after CeD seroconversion and compared these to values for individuals who did not develop CeD. In this way, we were able to elucidate differentially expressed genes (DEGs) and possible altered pathways in specific cell types found in PBMCs, as well as links with CeD-associated genetic risk loci. Such altered genes can pinpoint potential pre- and post-diagnostic markers of CeD.

## Materials and Methods

### Ethical Considerations and Study Design

The pediatric PreventCD cohort has been extensively described previously (17). Briefly, the at-risk children included in this study were newborns positive for HLA-DQ2 and/or -DQ8 from families in which at least one direct relative has been diagnosed with CeD. We excluded children with trisomy 21 or Turner syndrome and premature infants born at <36 weeks. The PreventCD samples used for this study were randomly selected based on donor disease status, sex and age. We used PBMC samples from 11 patients (confirmed diagnosis based on histopathology of small intestinal biopsy) before and after seroconversion for CeD and from 10 HLA genotype-, age- and sex-matched controls who did not develop CeD during the course of the PreventCD study. After selection of these samples, all experiments were performed in a blinded way. This study was conducted according to the guidelines laid down in the Declaration of Helsinki and it was approved by all medical ethics committees of the participating centers.

### Preparation of PBMCs

PBMCs were obtained from blood samples by gradient separation in Ficoll-Paque plus™, according to the manufacturer’s recommendations. Isolated PBMCs were cryopreserved in medium containing DMSO 10% and FCS 40% at -80°C, as described in Nazapour et al. (18), until use. Samples were thawed and prepared in batches of 10, consisting of 5 different donors sampled at two different timepoints. Upon thawing, cell concentration and viability were determined using a hemocytometer and Trypan Blue staining, respectively. Cells were then pooled into samples that contained 5 samples of different individuals (~1000 cells per donor).

### Library Preparation and Sequencing of scRNA

Single-cell cDNA libraries of PBMC pools were generated using the 10X Genomics platform following the manufacturer’s instructions (document CG00026) and as previously described (16). Briefly, each sample pool was loaded in one lane of Single Cell A Chip (10X Genomics, 120236), and the single-cell RNA was captured by beads, retro-transcribed into single-cell cDNA and amplified using the Single Cell 3’ Library & Gel Bead kit version 2 (10X Genomics, 120237) and the i7 Multiplex kit (10X Genomics, 120262). These libraries were sequenced according to 10X Genomics guidelines using the Novaseq 6000 S2 reagent kit (100 cycles) following a paired-end configuration. The final average sequencing depth was ~50 per cell. Raw sequencing data was processed using CellRanger v1.3 software with default settings. Sequencing reads from single cells were aligned to the hg19 reference genome.

### Demultiplexing of Donors, Processing and QC of scRNAseq Libraries

Donor DNA was genotyped using the Infinium GlobalScreeningArray-24v1.0. Next, we demultiplexed the data of the pooled samples based on their genotype using the Demuxlet algorithm, as previously described (16, 19). We applied the tool Opticall v.0.7.0 (20) to call genotypes using default settings. All samples had a call rate > 0.99. We did not identify any related individuals within the study group but did identify one individual of admixed ancestry who was kept in order to maximize our sample size. Cell doublets mapped to multiple donors were removed. We used the R package Seurat v.3.2 (21) to perform further analysis. We included cells expressing >200 and < 3000 unique genes and with < 15% mitochondrial counts for the downstream analysis.

### Clustering and Cell Type Identification of scRNAseq

The filtered single-cell dataset was analyzed to obtain the most variable genes across samples and cells, and the first 12 principle components were used for an unsupervised clustering with a resolution of 0.6 using the R package Seurat v.3.2 (21). Thereafter, to achieve an accurate annotation of cell cluster, we took a combined approach based on both well-annotated PBMC data and the known marker genes list of PBMC subsets. Specifically, we firstly applied a canonical correlation analysis to project the data onto a larger dataset of well-annotated PBMCs (16). The annotation of cell-types in the reference was then used to identify the cell populations in the query dataset. Second, the resulting cell types were validated by checking the expression of previously characterized marker genes of PBMC subsets (22).

### Differential Expression Analysis of scRNAseq

The generated data are from the following four categories of samples: data obtained from patients before and after seroconversion (CeD T0 and CeD T1, respectively) and from HLA genotype-, age- and sex-matched controls (CTR T0 and CTR T1). Using the “MAST” approach (23), we performed differential gene expression analyses comparing: 1) CeD T0 vs. CTR T1, 2) CeD T1 vs. CTR T1, 3) CeD T0 vs. CeD T1 and 4) CTR T0 vs. CTR T1. We only characterized transcripts that were expressed in > 10% of the cells of each subset and filtered in DEGs with an absolute log2FC > 0.25 and an adjusted p-value < 0.05. We then checked the data for the presence of 118 genes previously genetically associated with CeD and prioritized to play a possible causal role in CeD (24).

### Pathway Enrichment Analysis

The R package clusterProfiler v.3.14.3 was used to investigate whether the DE transcript sets were enriched for genes involved in specific pathways (P adjusted value < 0.05) (25).

### Cellular Communication Analysis

CellPhoneDB (26) was applied to the identified DEGs to infer immune ligand–receptor interactions in our dataset using the default parameters for the statistical analysis (receptor and ligand expressed in at least 10% of cells, 1000 permutations, FDR < 0.05). The R package ggplot2 was used to generate a heatmap visualizing the significance and the mean expression levels of these ligand–receptor gene pairs in different cellular subsets.

## Results

### Single-Cell Survey of PBMCs in CeD Context

**Figure 1:**
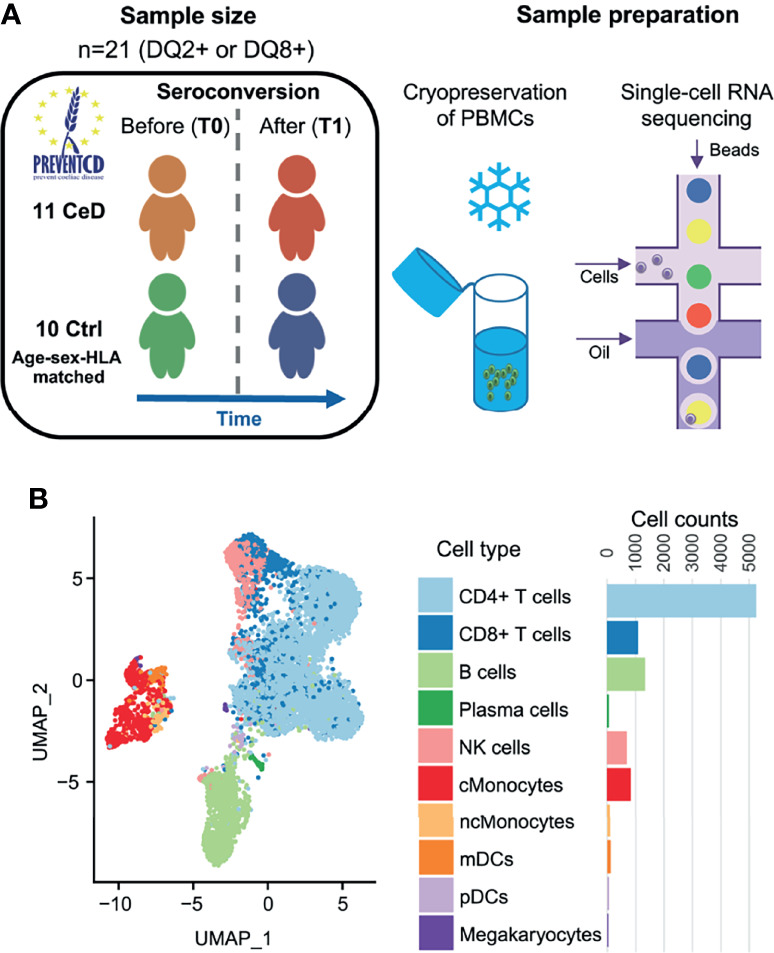
Single-cell survey of PBMCs in CeD context. **(A)** Study overview. The PreventCD participants used for this study were randomly selected based on disease status, sex, age and sample availability. We processed frozen PBMC samples from 11 different children before and after they were diagnosed with CeD and from 10 genotype-, age- and sex-matched controls who had not developed CeD. Single-cell RNA-seq analyses of these samples were measured using the 10X Genomics Chromium system. **(B)** UMAP plot of PBMCs. After QC, the transcriptomes of 9,559 PBMCs were obtained and classified into 10 cell types using a reference dataset (22). Each dot represents a single cell. Colors correspond to different cell-types. Cell counts per cell type are displayed on the right.

To study the differences at the onset of CeD at the transcriptomic level, we isolated PMBCs from 21 children included in the PreventCD cohort (17), who all were at high-risk of developing CeD. These samples were classified into cases or controls based on whether the donors developed CeD during the study, confirmed by histopathology of small intestinal biopsy. Samples (n=22) were collected from the same patients (n=11) before (T0) and after (T1) seroconversion for CeD as determined by the anti-TG2 antibody test (U/milliliters > 6). Control samples (n=20) from HLA genotype and sex matched donors (n=10) were taken at similar ages to T0 (n=10) and T1 (n=10) from CeD patients (**Figure 1A** and **Supplementary Table 1**). The average time between T0 and T1 was 16 months. In total, 19,663 single cells were profiled. After quality control by filtering based on possible doublets, the number of genes expressed (included cells with >200 and <3,000 genes) and low quality cells (included cells with <15% mitochondrial transcript reads)(**Supplementary Figure 1**), we retained 9,559 cells for subsequent analyses.

To annotate the cell types in our dataset, we applied both a standard clustering method and a reference-based approach using an external dataset of PBMCs from healthy controls that were processed in a similar manner (16). In total, we identified 10 cell subsets in the PBMC population: CD4+ T cells, CD8+ T cells, B cells, plasma cells, natural killer (NK) cells, classical monocytes (cMonocytes), non-classical monocytes, myeloid dendritic cells (mDCs), plasmacytoid dendritic cells (pDCs) and megakaryocytes (**Figure 1B**). The annotation of these subpopulations was validated by comparing the DEGs per cluster with known marker genes for each cell type (22).

After cell cluster annotation, we compared the cell frequencies of each subpopulation between disease states (cases vs controls) and between time points (T0/before seroconversion vs T1/after seroconversion) (**Supplementary Figure 2**). We tested whether the cell subset composition was significantly changed prior to or after CeD onset in CeD patients, excluding samples with fewer than 30 cells, and found that cell proportions were relatively stable in the blood of CeD patients compared to controls (**Supplementary Figure 2**). It is important to note that the main tissue affected in CeD is the small intestine and that the cell composition of this compartment will change upon CeD onset, for instance due to an expansion of lymphocytes (8, 27). However, such a compositional change does not seem to be reflected in the blood of our pediatric samples.

### Classification of DEGs Observed in CeD

**Figure 2:**
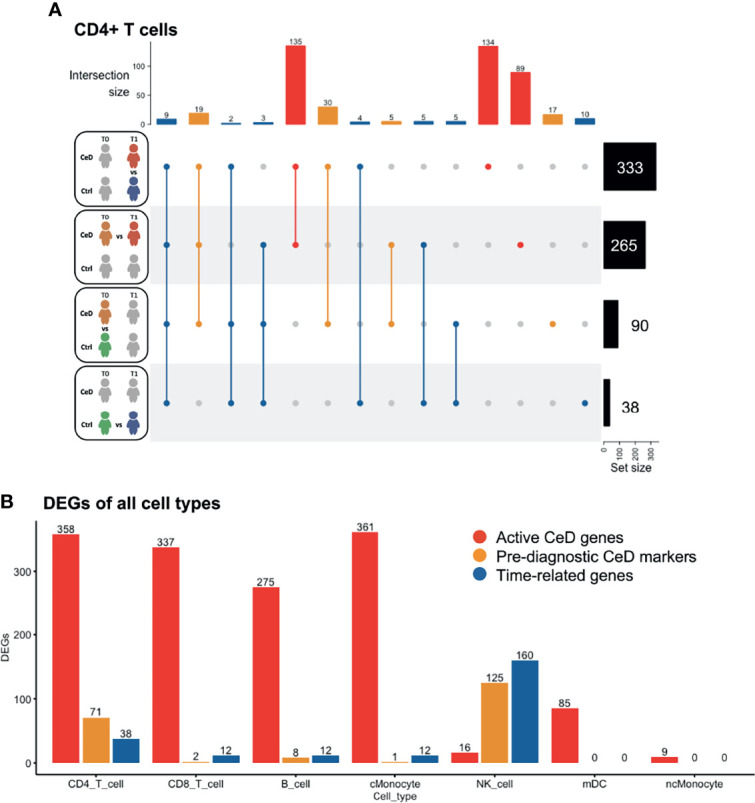
Differentially expressed genes classified in three groups. **(A)** Intersection of DEGs between different comparisons in CD4 T cells. The total number of DEGs per comparison is displayed on the right. Four comparisons were performed, as represented in the left part of the figure: CeD cases T1 vs. controls T1, CeD cases T0 vs. T1, CeD cases T0 vs. controls T0 and controls T0 vs. T1. The number of intersected DEG is shown at the top of the figure, in order of priority: time-related – all genes that showed overlap with comparison of controls T0 vs. T1, pre-diagnostic CeD markers – DEGs from the comparison of CeD T0 vs. controls T0, active CeD – DEGs from the comparison of CeD T1 vs. controls T1 and unique DEG when comparing the CeD T0 vs. T1. **(B)** An overview of the number of DEGs in different comparisons and cell types. On the y-axis, the number of DEGs (FDR < 0.05, absolute log2FC > 0.25) is displayed for active CeD (red), pre-diagnosis (yellow) and aging or time-related (blue). On the x-axis, the cell types are depicted.

To investigate the changes in gene expression profiles within each cell population, we performed differential expression analysis across disease status and across time of sampling (**Supplementary Figure 3**). DEGs were determined as specified in the Methods (FDR < 0.05 and absolute log2-fold change (log2FC) > 0.25) upon pooling each cell type in all samples from the same disease condition and sampling time, ending with four different comparisons: CeD T0 vs. T1, controls T0 vs. T1, CeD T0 vs. controls T0 and CeD T1 vs. controls T1 (as exemplified for CD4+ T cells in **Figure 2A**). We observed a varying number of DEGs in each cell type and category. CD4+ T cells showed the highest number of DEGs (n=467 genes), including 250 markers that were unique to only one of the comparisons, while the other DEGs were present in at least two of the comparisons (**Figure 2A**). The high number of DEGs in CD4+ T cells could be related to the increased power in this compartment as it is the most abundant cell type in our samples.

To better understand the role of DEGs in the context of CeD, we classified the DEGs into three categories: time-related genes, genes characteristic of active CeD and pre-diagnostic CeD markers. In the time-related gene category, we included all DEGs that overlapped or were unique to the T0 to T1 comparison within the controls. These genes are unlikely to be relevant for CeD and may be differentially expressed simply as a consequence of aging (these DEGs are indicated in blue in **Figure 2**). Active CeD genes were defined as DEGs from the comparison of cases vs. controls after seroconversion and from the comparison of T0 and T1 timepoints in cases, i.e. before seroconversion vs. after seroconversion (these DEGs are indicated in red in **Figures 2A, B** and **Supplementary Table 2**). Finally, we categorized the DEGs from the comparison of cases vs controls before seroconversion as pre-diagnostic CeD markers (indicated in yellow in **Figures 2A, B** and **Supplementary Table 3**).

**Figure 3:**
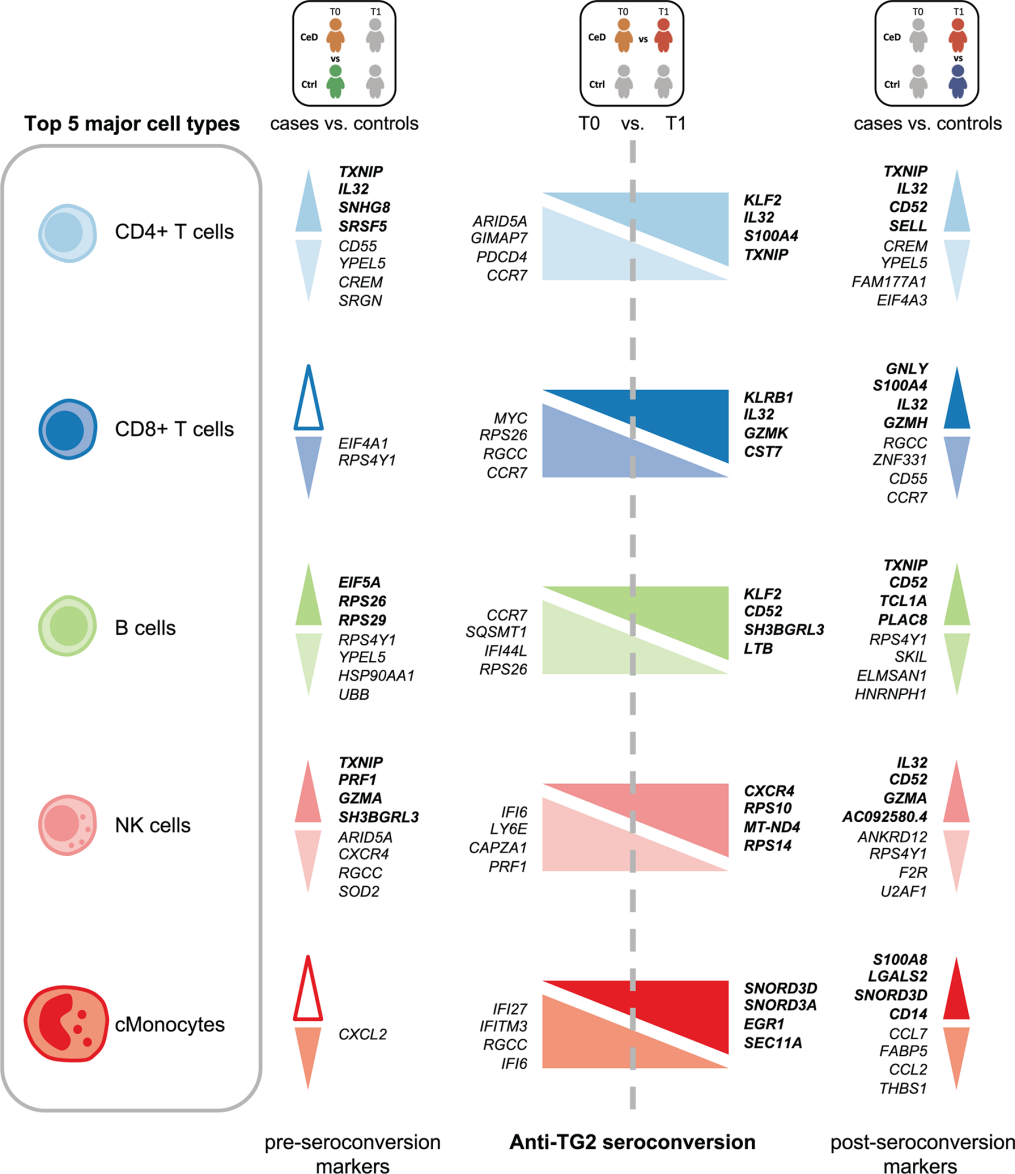
Top DEGs per cell type. Diagram of the top 4 DEGs (FDR < 0.05, absolute log2FC > 0.25) with the highest absolute log2FC value per cell type for the comparisons: CeD cases T0 vs. controls T0, CeD cases T0 vs. T1 and CeD cases T1 vs. controls T1. The top 5 most abundant cell types are depicted. Colors correspond to different cell-types. The upregulated genes are shown in bold font, whereas downregulated genes are in normal font. Color intensities of arrows indicate the direction of differential expression, where dark colors are for upregulation, light colors are for downregulation and white is used when no DEGs was found on that specific comparison.

Several cell populations showed distinct DEGs that were present before clinical onset of CeD (**Figure 2B** and **Supplementary Figure 3**). The top 4 DEGs for each comparison in the five most abundant cell types are depicted in **Figure 3**. These results demonstrate that CeD patients display a PBMC transcriptome profile that differs from that of healthy individuals. The existence of PBMC subsets with an altered transcriptome in pre-seroconversion conditions indicates that several biological processes may be altered in pediatric CeD patients even before they develop villous atrophy.

### Genes Differentially Expressed in CD4+ T Cells Are Related to Migration and Activation

The CD4+ T cell population exhibited the largest number of DEGs, including 358 DEGs classified as active CeD markers and 71 classified as pre-diagnostic markers (**Figure 2A** and **Supplementary Tables 2**, **3**). In **Supplementary Figures 4A, B**, we show that the changes were robust for each category. Some of the upregulated genes with the highest log2FC in the active CeD category included genes associated with T cell functions, such as *SELL* (also known as CD62L or l-selectin), *S100A4* and *CD52* (**Figure 3** and **Supplementary Figure 4A**). *SELL* and *S100A4* are involved in the migration of T cells (28, 29), whereas *CD52*, which is expressed in antigen-activated T cells, can induce suppression of other T cells (30, 31). Other top DEGs encode well-known transcription factors, including FOS (associated with a broad range of processes in the T cell activation) (32) and KLF2 (involved in cell motility) (33). The increased expression of these genes is therefore in line with a deregulation of CD4+ T cells in active CeD. In the pre-diagnostic marker category, the top genes were associated to diverse functions, such as thioredoxin interacting protein TXNIP (a regulator of cellular redox state whose function in CD4+ T cells seems to affect the proliferation of effector T cells) (34), and IL32 (previously associated with the progression of various inflammatory disorders including inflammatory bowel disease and gastric inflammation) (35, 36).

**Figure 4:**
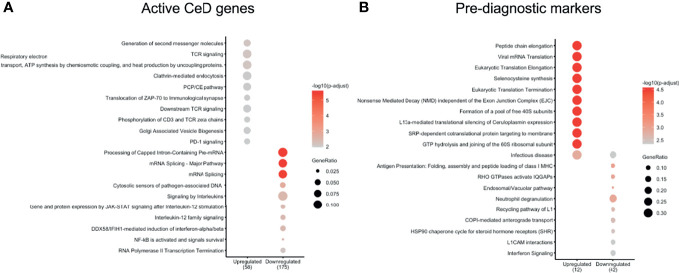
CD4 T cells show important DEGs associated with multiple immune-related pathways. Pathways enriched in **(A)** active CeD and **(B)** pre-diagnosis markers in CD4+ T cells identified using the Reactome database (P value < 0.05). Pathways are on the vertical axis. At the bottom is the direction of expression of the DEGs in CD4+ T cells. Numbers in brackets indicate the number of DEGs present in all enriched pathways. The size of dot indicates the ratio of the number of genes present in the gene set to the total number of genes used in each pathway.

We next explored which pathways were likely affected in each cell type by performing pathway enrichment analysis using the REACTOME database (**Supplementary Figures 5**–**9**). For CD4+ T cells, the genes upregulated after the seroconversion for CeD (**Figures 4A, B**) were significantly enriched for pathways related to immune-related functions (P adjusted value < 0.05) such as “TCR signaling” and “Generation of second messenger molecules” and mainly included genes such as *CD3G*, *CD3E* and *CD3D* (**Figure 4A** and **Supplementary Table 4**). In contrast, the downregulated DEGs were significantly enriched for transcripts involved in “Signaling by Interleukins” and “mRNA splicing”, amongst other pathways (**Figure 4A** and **Supplementary Table 4**). Some of the downregulated genes involved in interleukin signaling included important transcription factor–encoding genes such as *NFKB2* and *NFKBIA*. Both these genes code for proteins that have a regulatory function in NFκB signaling: *NFKB2* codes for p100, which can be processed into p52, a protein that act as a transcriptional repressor when it is in the homodimer form, and *NFKBIA* codes for IkBa, which inhibits NFκB signaling by retaining NFκB complexes in the cytosol (37). The downregulation of both inhibitory proteins may indicate an enhanced pro-inflammatory NFκB response. Additionally, some other genes found in the “Signaling by Interleukins” pathway, such as *SOCS1* and *SOCS3*, also code for suppressor proteins in the cytokine signaling (38), indicating a loss of inhibition of this pathway. NFκB pathway is known to be upregulated in CeD and plays an important role in the regulation of the inflammatory and immune response by controlling the expression of pro-inflammatory cytokines, adhesion proteins and enzymes in the small intestine mucosa (37, 39, 40).

In the case of the pre-diagnostic markers for CD4+ T cells, the upregulated genes included ribosomal proteins (e.g. *RPL39*, *RPL37*, *RPS28* and *RPS21*) that showed enrichments in pathways related to translation and “Infectious disease” (**Figure 4B** and **Supplementary Table 5**). This upregulation in the ribosomal proteins could be a response of CD4+ T cells that are already being stimulated in this early stage and could start preparing for the production of proteins. Downregulated genes were more abundant and included cytoskeleton genes (e.g. *ACTG1*, *TUBB2A* and *TUBA4A*) and HLA genes (e.g. *HLA-C* and *HLA-A*). Some of the enriched pathways of the downregulated genes were pathways related to antigen presentation and interferon signaling (**Figure 4B** and **Supplementary Table 5**).

### NK Cells of CeD Patients Express Potential Pre-Diagnostic Biomarkers for CeD

**Figure 5:**
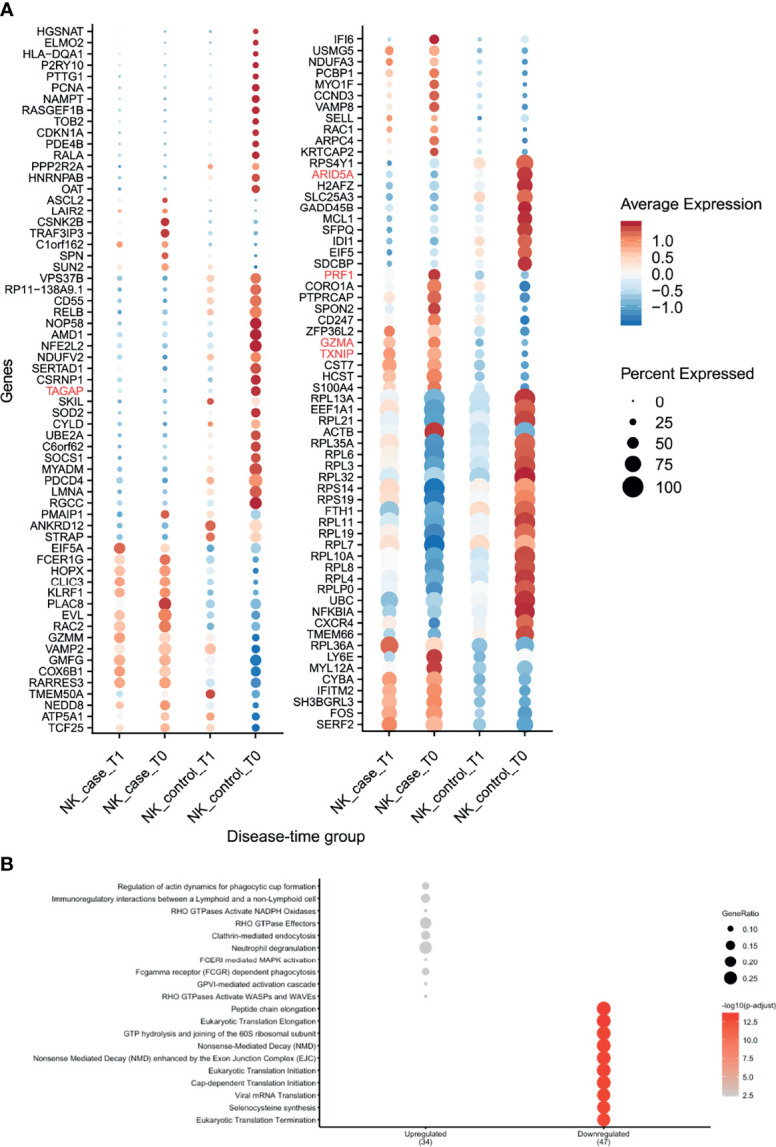
NK cells showed several differences in gene expression before seroconversion of CeD. **(A)** Dotplot shows the expression of DEGs classified as pre-diagnosis markers for NK cells. The average normalized expression is used to show how strongly the genes are expressed compared with the other groups, while the size of the dot indicates the percentage of cells within the cell type expressing that marker. Vertical axis shows the name of the genes, where red color is used for the top 5 DEGs. Horizontal axis indicates the condition and timepoint. **(B)** Pathway enrichment analysis for the pre-diagnosis markers in NK cells (similar to **Figure 3**).

NK cells showed the highest number of DEGs classified as pre-diagnostic markers (a total of 125) (**Figure 2A** and **Supplementary Tables 2**, **3**). The top genes with the highest log2FC were *TXNIP*, *PRF1* and *GZMA* (**Figure 3**, shown in red in **Figure 5A** and **Supplementary Table 3**), which are involved in NK cell activation, delivery of granzymes to target cells and cytotoxicity, respectively (41, 42). Pathway enrichment analysis showed that the upregulated pre-diagnostic markers are involved in interactions between lymphoid and non-lymphoid cells (e.g. *SELL*, *CD247* and *LAIR2*) and phagocytosis (e.g. *RAC1*, *ACTB* and *ARPC4*). Conversely, downregulated DEGs are enriched in “translation processes”, likely caused by the high number of ribosomal proteins that are downregulated (**Figure 5B** and **Supplementary Table 6**). One of the top downregulated genes was *ARID5A*, a ribosome-binding protein that has been previously associated with inflammatory autoimmune diseases (43). Overall, our results indicate that NK cells are already dysregulated before the onset of CeD.

### Cellular Communication Related to DE Genes in CeD

**Figure 6:**
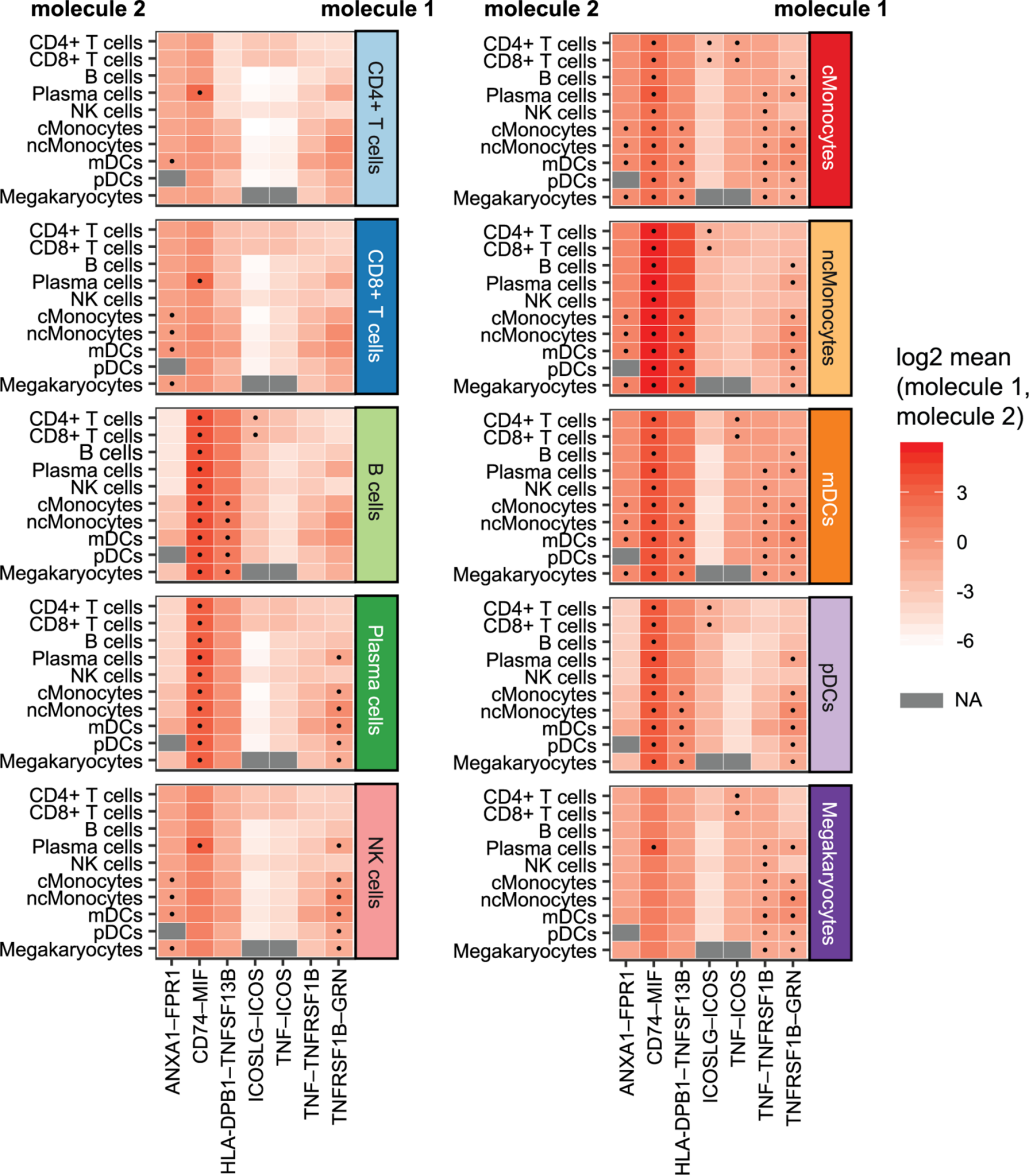
Cellular communication: potential interactions of DEGs in cells of CeD cases after diagnosis. Overview of selected ligand–receptor interactions determined using CellPhoneDB on the cells of CeD cases after seroconversion (T1). Horizontal axis shows the pairs of interacting molecule 1 and interacting molecule 2. Vertical axis indicates the cell types with identified interacting molecules: on the left are the cell types with the identified molecule 2, whereas cell types with the identified molecule 1 is on the right. The log2-transformed mean expression of interacting molecule 1 and interacting molecule 2 are indicated by color. Significant results (p-value < 0.05) are indicated with a dot. Grey color indicates NA results, usually occurring in cell types with few cells.

We investigated whether cell–cell interactions are altered in CeD based on responding genes by applying CellPhoneDB (26) to all DEGs in the cells from the CeD cases after seroconversion. CellPhoneDB is a tool that can be used to identify signaling crosstalk between immune cell subpopulations. This analysis identified seven significant ligand–receptor pairs (receptor and ligand expressed in at least 10% of cells, 1000 permutations, FDR < 0.05) expressed among the different cell subpopulations (**Figure 6**). These include potential CD74–MIF signaling interactions from possible antigen-presenting cells (such as Monocytes, DCs and B cells expressing CD74) towards adaptive immune cells (including CD4+ T cells, CD8+ T cells and plasma cells expressing MIF). Moreover, the ligand–receptor pairs ANXA1–FPR1, HLA-DPB1–TNFSF13B, TNF–TNFRSF1B and TNFRSF1B–GRN were found mainly between monocytes, while ICOSLG–ICOS and TNF–ICOS were mainly found between monocytes and CD4+ T cells. Thus, the cell–cell interactions we identified may be disrupted as a consequence of the differential expression of these genes during the onset of CeD.

### Risk-Associated DEGs Are Mainly Found in T and NK Cells

**Figure 7:**
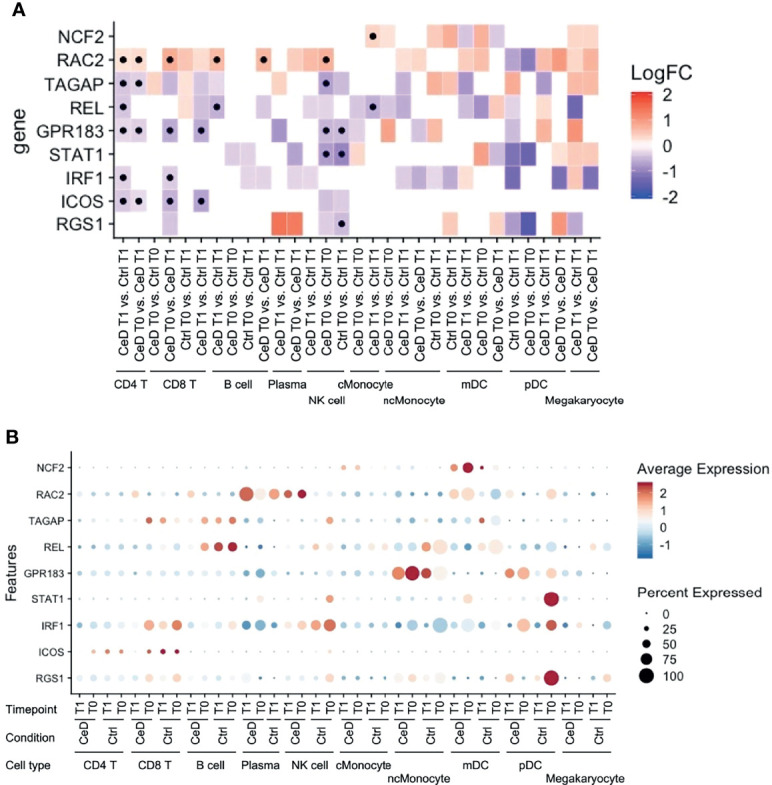
Expression pattern of GWAS-associated CeD genes. **(A)** Heatmap with the log2FC of all intersected DEGs with the 118 risk genes prioritized from CeD loci from v. d. Graaf et al. (24). Vertical axis shows the name of the genes. Horizontal axis corresponds to the different comparisons performed per cell type, timepoint and condition. Black dots indicate whether the gene is significant (adjusted p-value < 0.05). **(B)** Dotplot of the intersected DEGs to the prioritized genes to show the relative average normalized expression of the genes in the different groups and cell types. (similar to **Figure 5**).

Like inflammation and environmental factors, genetic risk factors may also assert effects on gene expression in the context of CeD. To date, 43 genetic risk factors have been identified for CeD, excluding the HLA locus, which accounts for approximately 40% of disease risk (44, 45). In order to detect the cell types that are most likely affected by CeD genetic factors, we intersected our DEGs with the CeD risk/causal genes prioritized in another study (24). Among 118 genes potentially causal in CeD, nine (*NCF2, RAC2, TAGAP, REL, GPR183, STAT1, IRF1, ICOS* and *RGS1*) were significantly differentially expressed in at least one comparison in our data (**Figure 7A**). Eight of these were observed in both T cells and NK cells (**Figures 7A, B**), suggesting the importance of the genetic background in these cell types in CeD context. One example here is *TAGAP*, which is a marker for active CeD in CD4+ T cells and a pre-diagnostic marker in NK cells. A potential role for genetic risk factors associated with CeD in the regulation of *TAGAP* expression is partly supported by public data, which shows that CeD risk allele T (rs1738074) [Z-OR=0.15, P value= 1.04e-15] (46) leads to a downregulation of *TAGAP* in blood [Z= -9.01, P value= 2.0419e-19] (47). Indeed, eight of the nine genes (*NCF2, TAGAP, REL, GPR183, STAT1, IRF1, ICOS* and *RGS1*) are downregulated in cases compared to controls and/or after developing CeD.

## Discussion

Using scRNAseq of the PBMC fraction of blood, we identified CeD biomarkers present in prospective and active CeD individuals. Per cell type, we classified predictive and diagnostic biomarkers based on the time when specific transcripts were differentially expressed. Moreover, we found potential CeD-associated transcriptional changes that may affect cell–cell communication and/or interactions that occur in the PBMC subsets. Finally, we also identified markers that had been genetically associated to CeD in previous GWAS and eQTL analyses. Altogether, our results pinpoint genes and pathways altered in CeD that are potentially diagnostic markers for CeD.

Based on our scRNAseq data, the PBMCs clustered in 10 cellular subsets. Overall, we found relatively stable cell proportions for each cell type when comparing the four experimental groups (CeD patients before seroconversion (T0) and after seroconversion (T1) and samples taken at two timepoints of control individuals age-matched for both patient groups). Previously, van Unen et al. showed that disease-specific leukocytes reside mainly in the affected organ and are hardly detectable in PBMC (48). Other studies have reported variations in the proportions of specific cell populations in CeD blood samples, including of circulating gluten-specific T cells (49, 50). Although we were able to successfully classify the major cellular lineages in PBMCs, we could not assess the presence of this rare subset because it is very scarce (0.1-1000 gluten-specific T cells per 10^6^ CD4+ T cells (49, 50). However, our results show that we can find useful CeD biomarkers even within the ‘common PBMC lineages’.

Our results agree with literature describing the importance of CD4+ T cells in CeD, since this cell type compartment is known to have a critical role in the CeD immunopathogenesis (15, 51). We identified the highest number of DEGs in CD4+ T cells. Most of these DEGs were biomarkers for active CeD, but there were also some potential pre-diagnostic biomarkers. The pool of upregulated ‘active CeD’ biomarkers showed genes associated with T cell activation (e.g. *CD52*) and migration (e.g. *SELL* and *S100A4*). *CD52* acts as the effector and marker molecule of specific CD4+ T cells that modulate the activation of other T cells (30). CD52 is also used as target with anti-CD52 gene to reduce the severity of associated symptoms to multiple sclerosis (52). However, the role of *CD52* in CeD remains uncertain. In contrast, the downregulated genes showed an enrichment for interleukin signaling and NFκB pathways, including key inhibitory genes such as *NFKB2* and *NFKBIA*, thus suggesting an upregulation of the NFκB pathway. Although the interleukin signaling is a major feature of active CD4+ T cells in CeD pathophysiology (53), the number of differentially expressed interleukins that we detected was low, but this could be due to the limit of detection of scRNAseq for interleukin genes or by the fact that the CD4+ T cells that contribute to CeD reside predominantly in the small intestine, while very little of them are in circulation.

The NK cells were characterized by a high number (n=125) of pre-diagnostic biomarkers. Many of these genes relate to cytotoxicity (e.g. *GZMA, GZMM, PRF1* and *TXNIP*). These genes are of great interest for diagnostic purposes because their levels are raised in individuals who later develop CeD and they are expressed in a high fraction of the NK cells (**Figure 5A**). Interestingly, one of these genes is *TAGAP*, whose locus has been genetically associated to several infectious and autoimmune diseases, including candidemia (54), multiple sclerosis (55) and CeD (24). We find *TAGAP* to be differentially expressed in both the CD4+ T cell and NK subsets. However, before seroconversion it is differentially expressed in NK cells, while after seroconversion it is differentially expressed in CD4+ T cells, a shift that demonstrates that cell type should be taken into consideration when assessing biomarkers. The function of *TAGAP* in CD4+ T cells is related to activation, and it plays an important role in Th17-cell differentiation (56, 57). In NK cells, *TAGAP’s* function is unclear. Additionally, although another study suggested *TAGAP* as a pre-diagnostic marker (13), the changes in expression levels of this gene are non-concordant between our study and that of Galatola et al. (13), which may indicate that NK cells are not the main source of the *TAGAP* levels observed by Galatola et al. (13). Although we found a high number of pre-diagnostic markers in NK cells, the role of these cells in CeD remains uncertain, since the main lymphocytes known to contribute to CeD onset are CD4+ T cells and intraepithelial lymphocytes in the small intestine. However, novel findings about the possible role of NK cells in autoimmune diseases have emerged in recent years. In multiple sclerosis, genetic risk loci have been found to be more enriched in open chromatin regions of NK cells (58). For type 1 diabetes, it was reported that specific peripheral NK cell subsets may serve as potential candidate biomarkers of disease progression (59). In the context of CeD, Type 1 innate lymphoid cells, which share a similar cytotoxic profile and several markers with conventional NK cells, have been demonstrated to be altered in active CeD (60, 61). Future experiments to confirm a possible early role in CeD onset for the NK cell compartment in both PBMCs and the duodenum are therefore warranted.

In addition, we highlight seven ligand–receptor pairs in DEGs that might be affected in CeD: ANXA1–FPR1, CD74–MIF, HLA-DPB1–TNFSF13B, ICOSLG–ICOS, TNF–ICOS, TNF–TNFRSF1B and TNFRSF1B–GRN. Among these, four pairs are involved in TNF or TNFRSF1B (also known as TNFR2) signaling. TNF encodes a cytokine known to mediate cell signaling and plays an important role in innate immunity, whereas TNFRSF1B encodes for its receptor (37, 40, 62, 63). This finding, taken together with the downregulation of genes such as *NFKB2* and *NFKBIA* that inhibit the NFκB pathway, would support our finding that this pathway is activated in CeD context.

We found that cross-talk of myeloid cells (monocytes and dendritic cells) by TNF is potentially affected in active CeD and that B cells may also react to TNF. This is consistent with the known pathogenic function of the TNF pathway in CeD (53). CD4+ T cells’ interactions with TNF *via* ICOS are also probably affected, which could imply a role for ICOS+ CD4+ T cells in the dysregulation of CeD. Similarly, the interaction between ICOS (downregulated in CD4+ T cells and CD8+ T cells) and ICOSLG (downregulated in B cells) was found to be potentially affected between CD4+ and CD8+ T cells with B cells, monocytes and dendritic cells in CeD context. ICOSLG-ICOS interaction is implicated in multiple immune processes, such as the establishment of CD8+ tissue resident T cells in intestine (64). Altogether, these interactions point to the importance of ICOS interactions, which have been found to have relevance in multiple autoimmune diseases (65–67).

Nine potential CeD risk genes [of the 118 genes prioritized CeD risk genes by van der Graaf et al. (24)] were significantly differentially expressed in at least one comparison in our data: *NCF2, RAC2, TAGAP, REL GPR183, STAT1, IRF1, ICOS* and *RGS1*. Eight of these genes (*RAC2, TAGAP, REL GPR183, STAT1, IRF1, ICOS* and *RGS1*) were found in T cells and NK cells, but only *RAC2* was upregulated in the CeD condition. Loss-of-function of *RAC2* causes immunodeficiency (68), while upregulation of this gene is a marker of poor clinical prognosis of certain carcinomas (69).

Counterintuitively, several of the genes we see downregulated, *TAGAP, REL,* and *GPR183,* have been reported to be upregulated in gluten-specific CD4+ T cells after activation with antiCD3/CD28 or under active CeD conditions, while levels of *STAT1* and *IRF1* are reportedly higher in biopsies isolated from CeD cases (70, 71). We speculate that these contrasting observations might reflect the fact that we are looking at a tissue (blood) in which the pathological symptoms of CeD are not most dominant, and this may cause differences between our observations and those made in inflamed tissues (intestine). Moreover, gluten-specific CD4+ T cells are only a small subset of the CD4+ T cells in circulation (49, 50). Of note, genes differentially expressed in one cell type might have the highest expression level in another cell type (**Figure 7B**). For example, *NCF2* showed the highest expression level in mDC, but the difference between cases and controls was observed in cMonocytes. However, the tissue/cell type where the risk genes are highly expressed is usually considered as the causal cell type. Our observations highlight the importance of assessing all cell types, including those where the risk genes may have a relatively low expression level.

We acknowledge that our study has some limitations. For instance, clustering distinct populations of CD8+ T cells and NK cells is not straightforward, potentially hampering the identification of NK cell–specific prediagnostic markers. Moreover, our study has a small overall sample size, which may affect the robustness of our observations and study detection power. Additionally, while the included controls did not convert to active CeD within a time frame of at least 10 years, conversion is still possible as these were all individuals at high risk for CeD. Our findings are limited to the development of CeD at early life, which may differ from ongoing pathogenesis at later ages. Hence, further studies performed on cohorts that follow adult high risk CeD individuals may allow for a better understanding of CeD onset. Lastly, our results only pertain to children at high risk of developing CeD. To understand if children at high risk of CeD onset exhibit different gene expression patterns than children without genetic and familial risk for CeD would have required taking control samples from age-matched children from the general population, which could not be done due to legal and ethical concerns.

In conclusion, we used scRNAseq to analyze the immune cells of children at high risk of developing CeD, before and after seroconversion. We validated previous associations of cell types and genes to CeD onset and obtained new insights about the changes in immune cells before and after CeD onset. In CD4+ T cells, we found an upregulation of genes associated to migration (*SELL*, *S100A4*) and activation (*CD52*) and a downregulation of key inhibitory genes of the NFκB pathway (*NFKB2* and *NFKBIA*). Some possible interactions between immune cell compartments were potentially affected, such as that between ICOSLG and its receptor ICOS. This finding further highlights the contribution of *ICOS*, a CeD GWAS associated gene, in the development of CeD. Finally, we identified potential novel biomarkers for the diagnosis of CeD, especially in the NK cell compartment (*GZMA, GZMM, PRF1*, *TXNIP* and *TAGAP*), that can be detected before the duodenal damage. Such biomarkers might provide potential alternatives to biopsy-dependent diagnosis of CeD patients.

## Data Availability Statement

The sequencing reads and genotypes used in this article are not readily available due to ethical and privacy restrictions. Requests to access the datasets should be directed to the corresponding author. The scRNAseq count matrix and metadata associated to cell-barcodes are available as **Supplementary Material**.

## Ethics Statement

The studies involving human participants were reviewed and approved by Commissie Medische Ethiek LUMC, Leiden, the Netherlands; Institutional Research Ethics Committee, Heim Pal Childrens Hospital, Budapest, Hungary; and Ethikkomimision LMU, Munich, Germany. Written informed consent to participate in this study was provided by the participants’ legal guardian/next of kin.

## Author Contributions

AR-S, RM, and YK-W performed wet-lab experiments. LM, SK, and IK-S provided samples. AR-S, XC, SW, IJ, and YL conceived and wrote the manuscript. XC and AR-S performed the statistical analyses. IJ, YL, SW, LM, SK, IK-S, RT, and CW supervised and edited the manuscript. All authors contributed to the article and approved the submitted version.

## Funding

AR-S is supported by a CONACYT-I2T2 scholarship (no. 459192). X.C. was supported by Chinese Scholarship Council (201706040081). CW is supported by an NWO Spinoza prize (NWO SPI 92–266). IK-S was supported by grants NKFI 120392 from the Hungarian National Research, Development and Innovation Fund and GINOP-2.3.2-15-2016-00015 co-financed by the European Union and the Hungarian State. SW and CW are supported by the Netherlands Organ-on-Chip Initiative, an NWO Gravitation project (024.003.001) funded by the Ministry of Education, Culture and Science of the government of the Netherlands. IJ is supported by a Rosalind Franklin Fellowship from the University of Groningen and a Netherlands Organization for Scientific Research (NWO) VIDI grant (no. 016.171.047). This work was supported by a ZonMW-OffRoad grant (91215206) to YL. YL is also supported by a Radboud University Medical Centre Hypatia Grant (2018) and an ERC starting grant (948207). PreventCD consortium is supported by grants from the European Commission (FP6-2005-FOOD-4B-36383–PREVENTCD), the Azrieli Foundation, Deutsche Zöliakie Gesellschaft, Eurospital, Fondazione Celiachia, Fria Bröd Sweden, Instituto de Salud Carlos III, Spanish Society for Pediatric Gastroenterology, Hepatology, and Nutrition, Komitet Badañ Naukowych (1715/B/P01/2008/34), Fundacja Nutricia (1W44/FNUT3/2013), Hungarian Scientific Research Funds (OTKA101788 and TAMOP 2.2.11/1/KONV-20 12-0023), Stichting Coeliakie Onderzoek Nederland (STICOON), Thermo Fisher Scientific, and the European Society for Pediatric Gastroenterology, Hepatology, and Nutrition (ESPGHAN).

## Conflict of Interest

The authors declare that the research was conducted in the absence of any commercial or financial relationships that could be construed as a potential conflict of interest.

## Publisher’s Note

All claims expressed in this article are solely those of the authors and do not necessarily represent those of their affiliated organizations, or those of the publisher, the editors and the reviewers. Any product that may be evaluated in this article, or claim that may be made by its manufacturer, is not guaranteed or endorsed by the publisher.

## Supplementary Material

Supplementary Data Sheet 1 Metadata of scRNAseq per cell-barcode.

Supplementary Data Sheet 2 Matrix of scRNAseq raw counts of genes per cell-barcode.

Supplementary Figure 1 QC of scRNAseq data.

Supplementary Figure 2 Percentages of cell types among cases and controls per timepoint.

Supplementary Figure 3 Intersection and classification on all DE genes for each cell type and comparison.

Supplementary Figure 4 DE of active CeD genes and pre-diagnostic markers of CD4+ T cells.

Supplementary Figure 5 Pathway enrichment analysis in CD8+ T cells.

Supplementary Figure 6 Pathway enrichment analysis in B cells.

Supplementary Figure 7 Pathway enrichment analysis in classical Monocytes.

Supplementary Figure 8 Pathway enrichment analysis in non-classical Monocytes.

Supplementary Figure 9 Pathway enrichment analysis in Dendritic cells.

Supplementary Table 1 Sample sheet and sequencing details.

Supplementary Table 2 List of DEG classified as active CeD markers per cell-type.

Supplementary Table 3 List of DEG classified as pre-diagnostic CeD markers per cell-type.

Supplementary Table 4 Enriched pathways of active CeD genes in CD4+ T cells.

Supplementary Table 5 Enriched pathways of pre-diagnostic CeD genes in CD4+ T cells.

Supplementary Table 6 Enriched pathways of pre-diagnostic CeD genes in NK cells.

Supplementary Table 7 List of scRNAseq gene markers used in the cell-type identification.
