# Supplementary material for: Single-Cell RNA Sequencing of Peripheral Blood Mononuclear Cells From Pediatric Coeliac Disease Patients Suggests Potential Pre-Seroconversion Markers
Source: Front Immunol. 2022 Mar 15;13:843086. doi: 10.3389/fimmu.2022.843086 (PMC8964997; doi:10.3389/fimmu.2022.843086)
Supplement: Supplementary Figure 1 — QC of scRNAseq data. [file Image_1.pdf]

Supplementary Figures

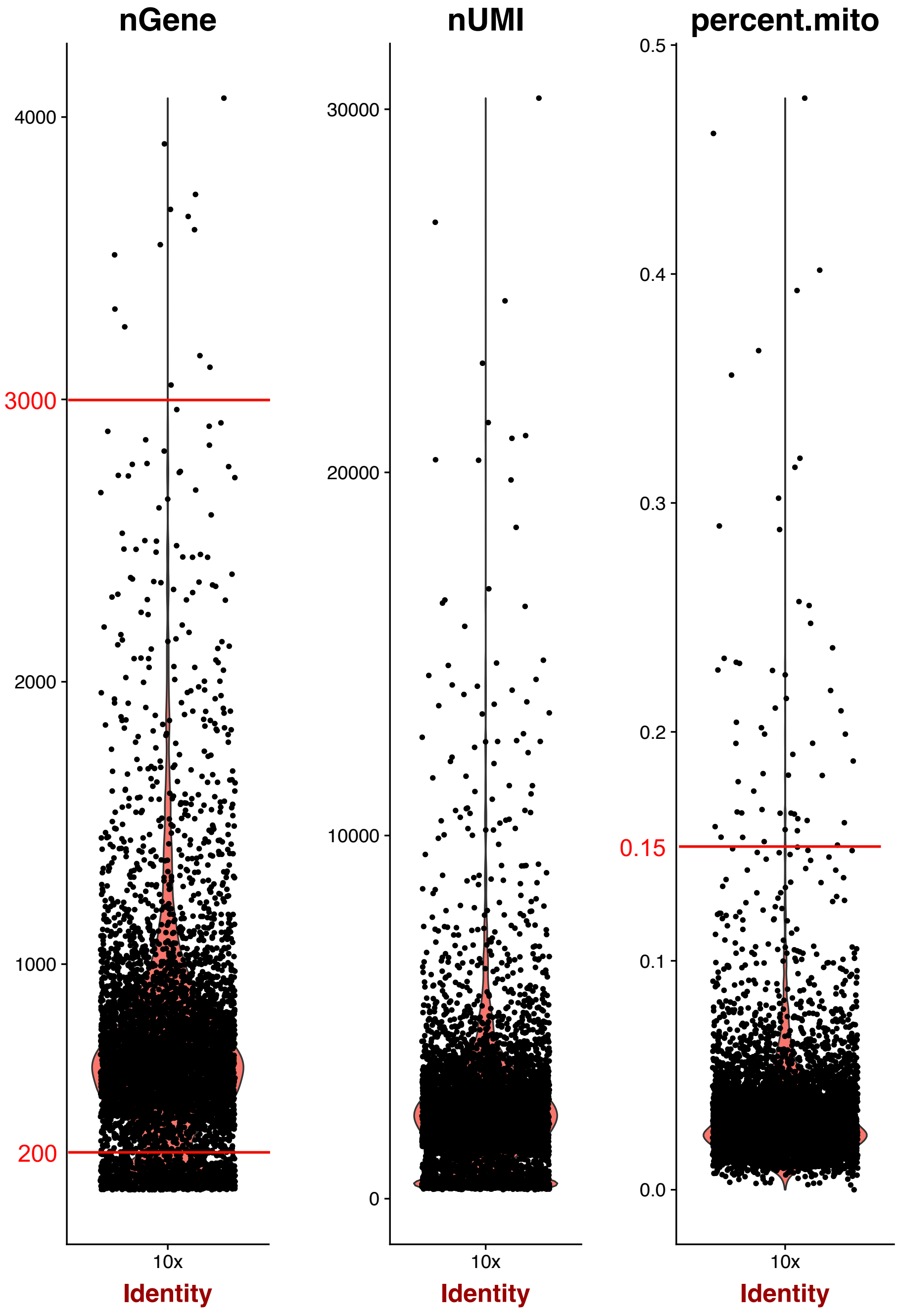

Supplementary figure 1. QC of scRNAseq data. Dotplots for the number of unique genes (nGene), number of unique molecular identifier (nUMI) and percentage of mitochondrial genes from total expression for each single-cell. Each dot represents a single cell. Values showed in red are the thresholds used to filter bad quality cells as described in Materials and Methods section.

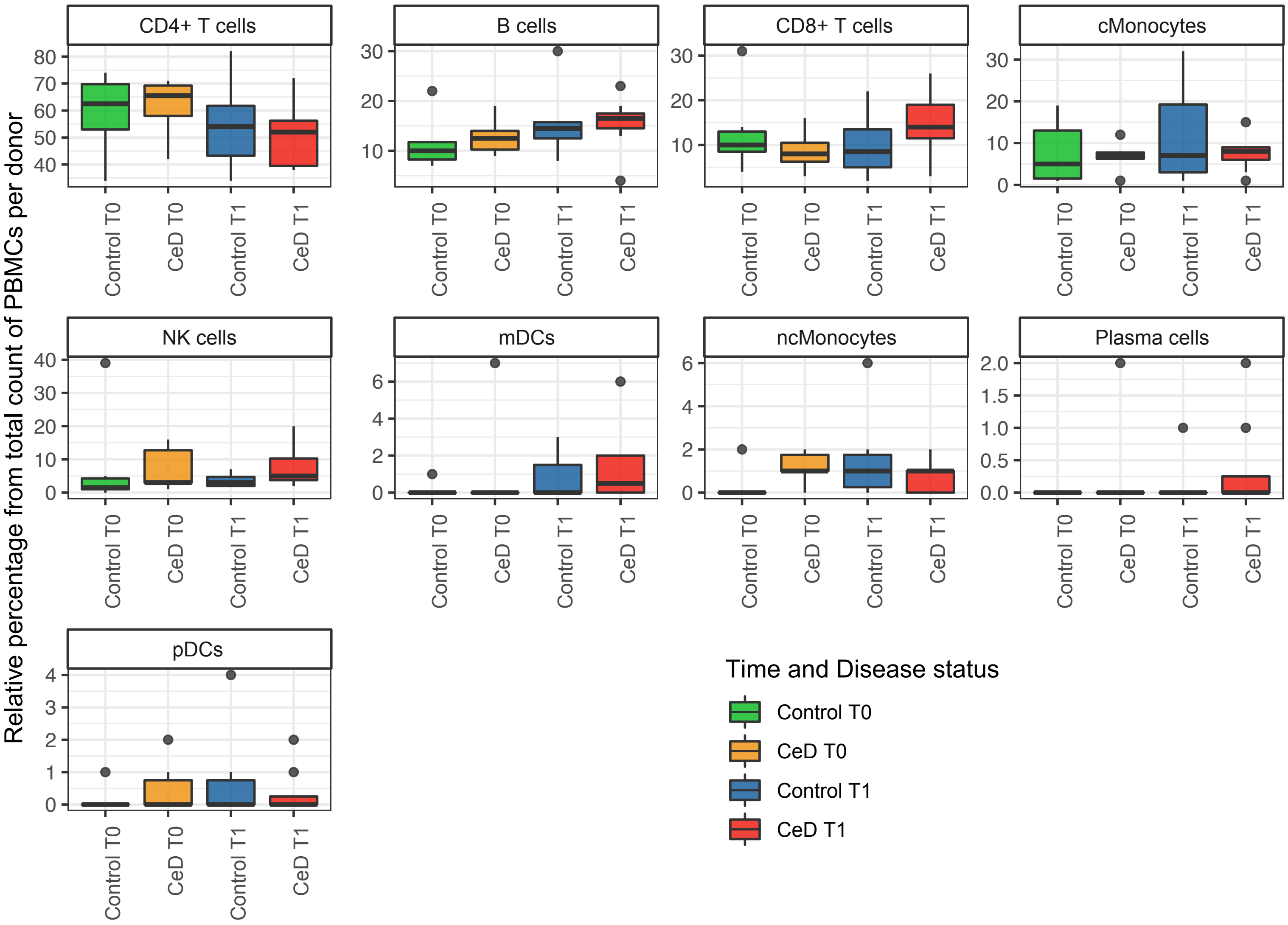

Supplementary figure 2. Percentages of cell types among cases and controls per timepoint. Cell proportion comparison across conditions and timepoints. The cell proportions of the majority of cell types distribute similarly across states of disease. Samples were grouped into four different conditions: patients before (CeD T0) and after seroconversion (CeD T1) and matched controls at the same sampling time points (Control T0 and Control T1). The comparison of cell proportions between conditions was done using a Student's t-test with Control T1 used as baseline.

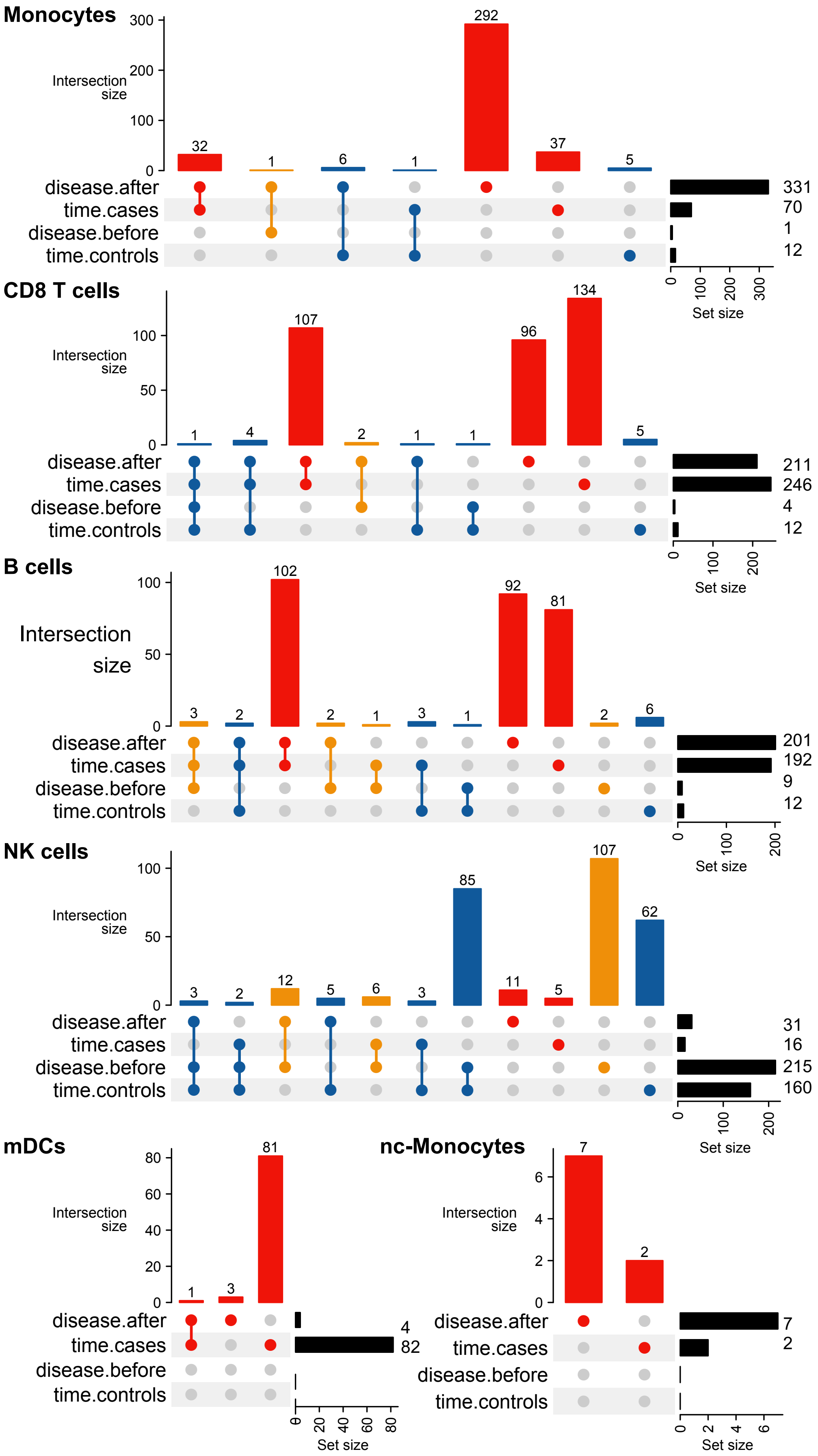

Supplementary figure 3. Intersection and classification on all DE genes for each cell type and comparison. Visualization using UpSet plots of the intersection of the multiple DEGs obtained from the different comparisons between the disease vs. controls before and after diagnosis, per cell type. As described in Figure 2A, the total number of DEGs per comparison is displayed on the right-hand side. The left part of the figure shows the four comparisons performed: CeD cases T1 vs. controls T1, CeD cases T0 vs. T1, CeD cases T0 vs. controls T0 and controls T0 vs. T1. Colors indicate active CeD genes (red), time-related genes (blue) and pre-diagnostic markers (yellow).

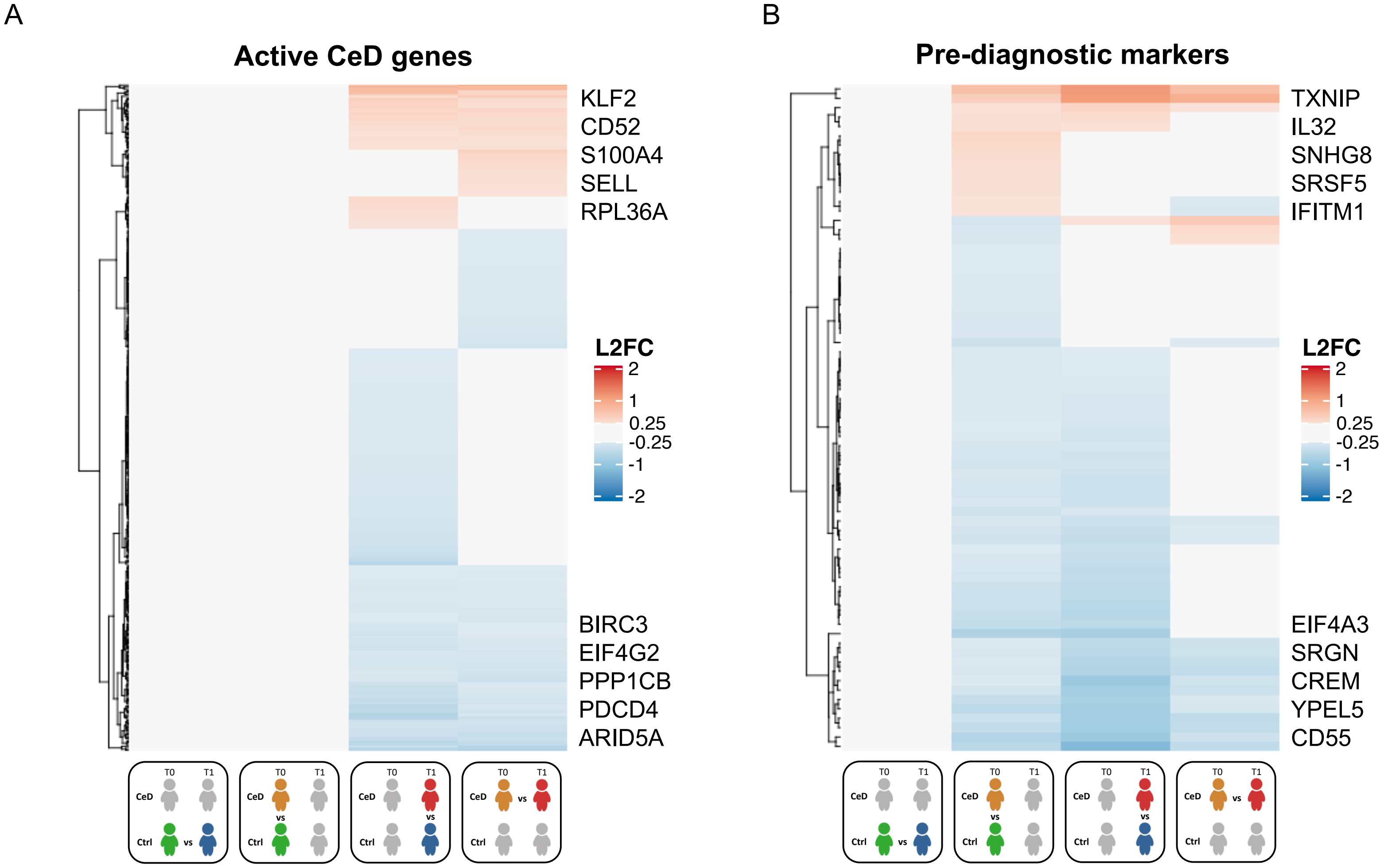

Supplementary figure 4. DE of active CeD genes and pre-diagnostic markers of CD4+ T cells. Heatmaps of the log2FC of **(A)** active CeD and **(B)** pre-diagnosis markers found in CD4+ T cells. Each row corresponds to a DEG, while the columns are the comparisons used in the analysis. At the right of the heatmaps are the top five genes with the highest absolute log2FC value for the upregulated (top) or downregulated (bottom) genes.

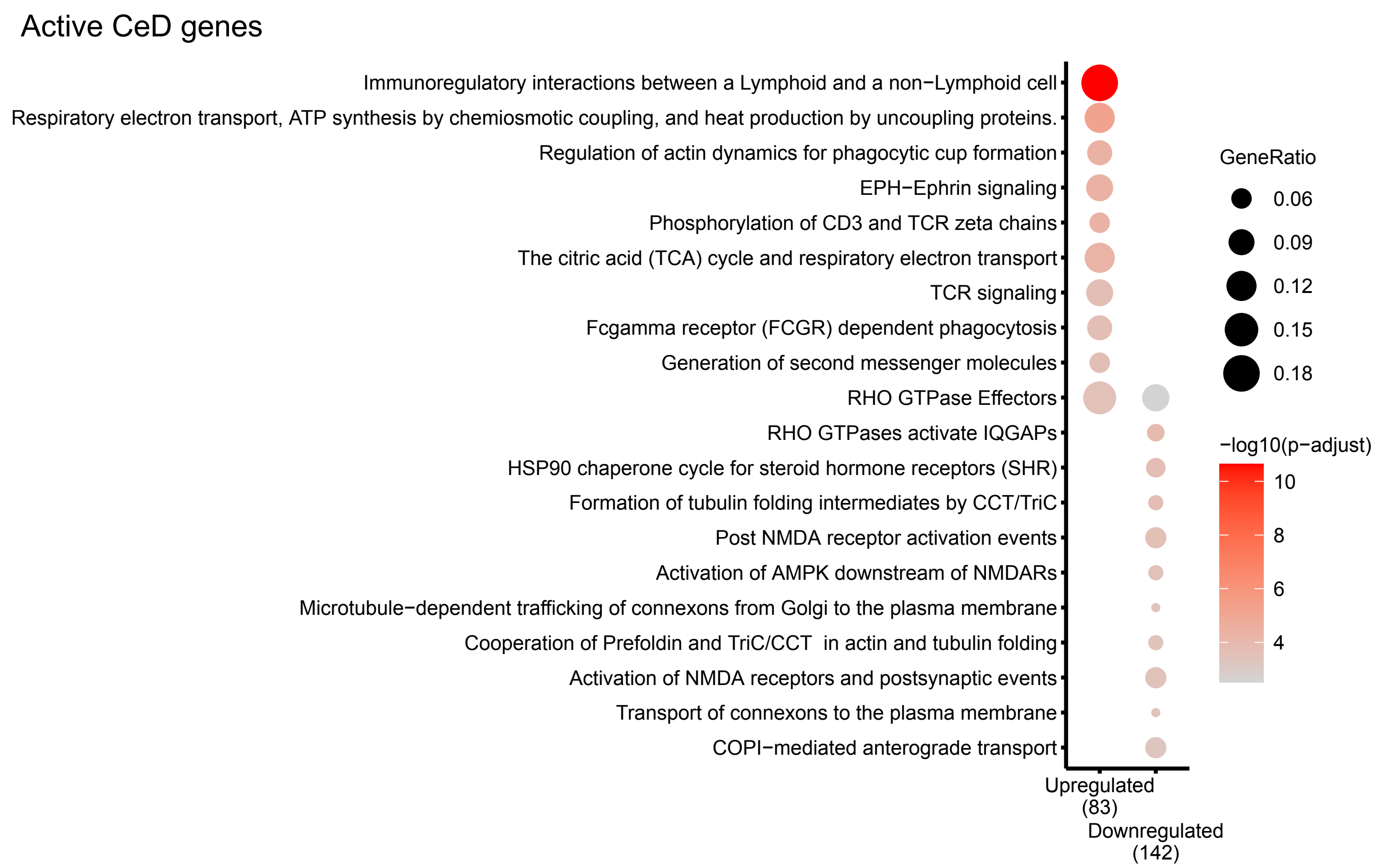

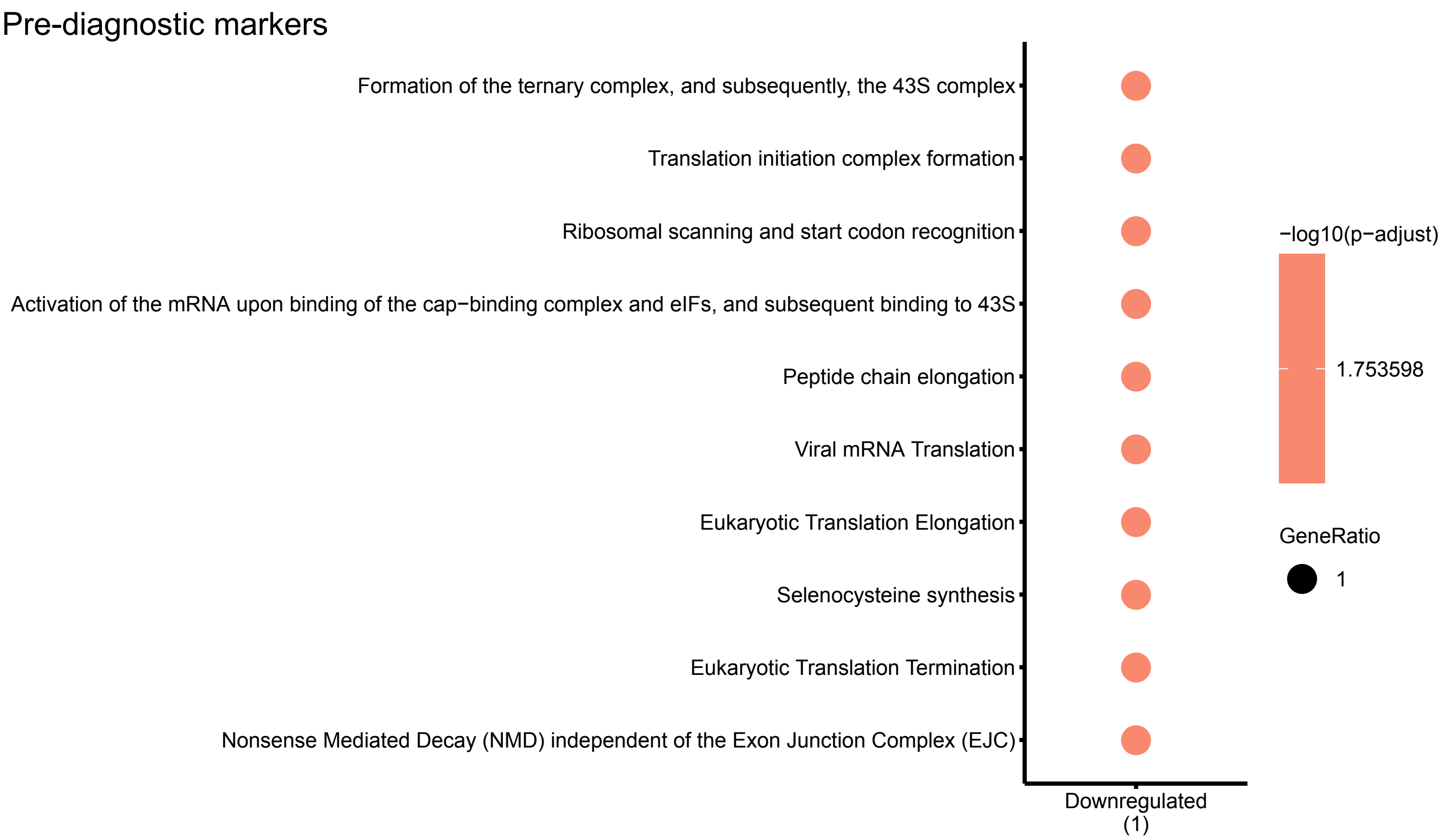

Supplementary figure 5. Pathway enrichment analysis in CD8+ T cells. Pathways enriched in active CeD and pre-diagnosis markers in CD8+ T cells identified using the Reactome database (p-value < 0.05). Pathways are on the vertical axis. At the bottom is the direction of expression of the DEGs in CD8+ T cells. Numbers in brackets indicate the number of DEGs present in all enriched pathways. The size of dot indicates the ratio of the number of genes present in the gene set to the total number of genes used in each pathway.

Active CeD genes

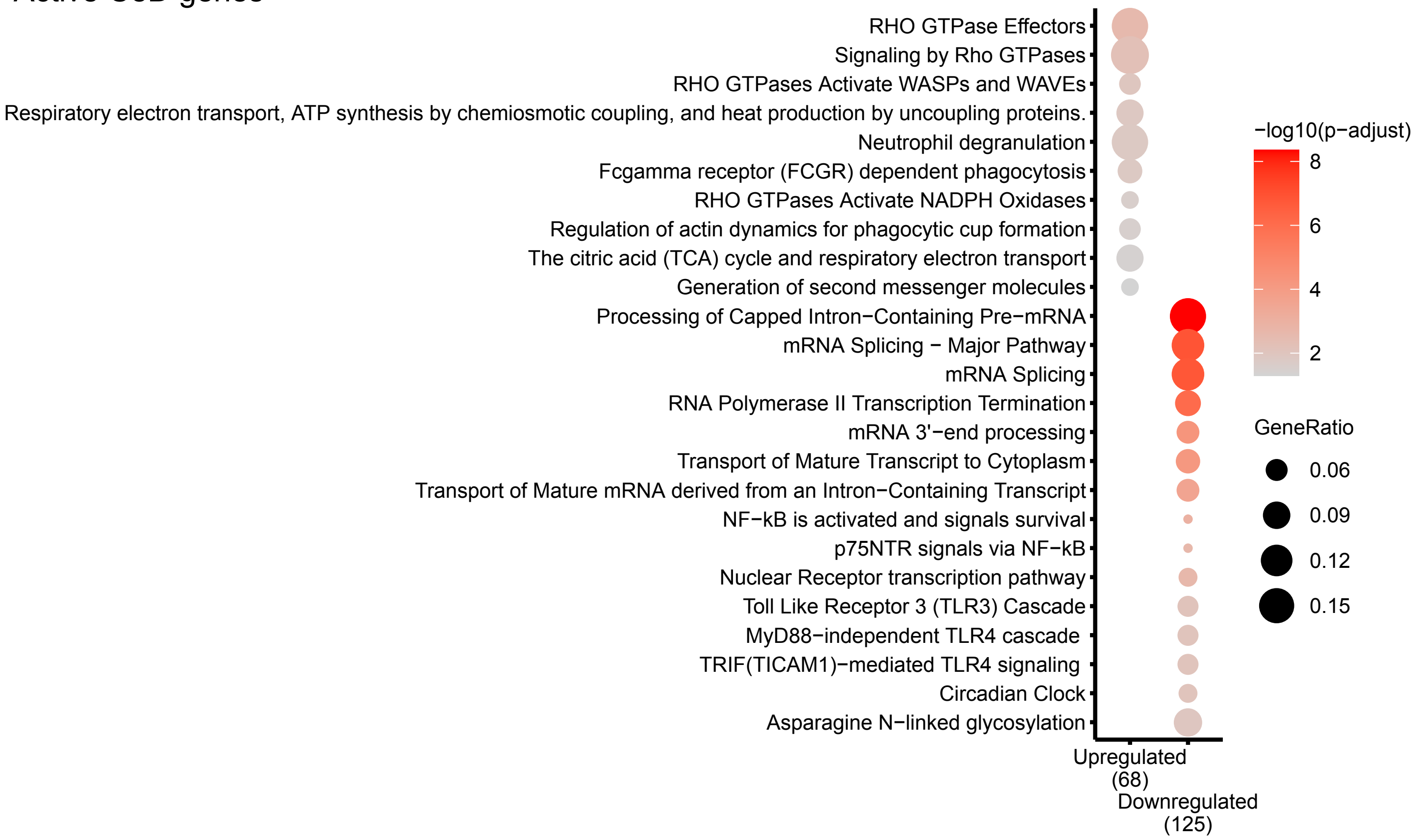

Pre-diagnostic markers

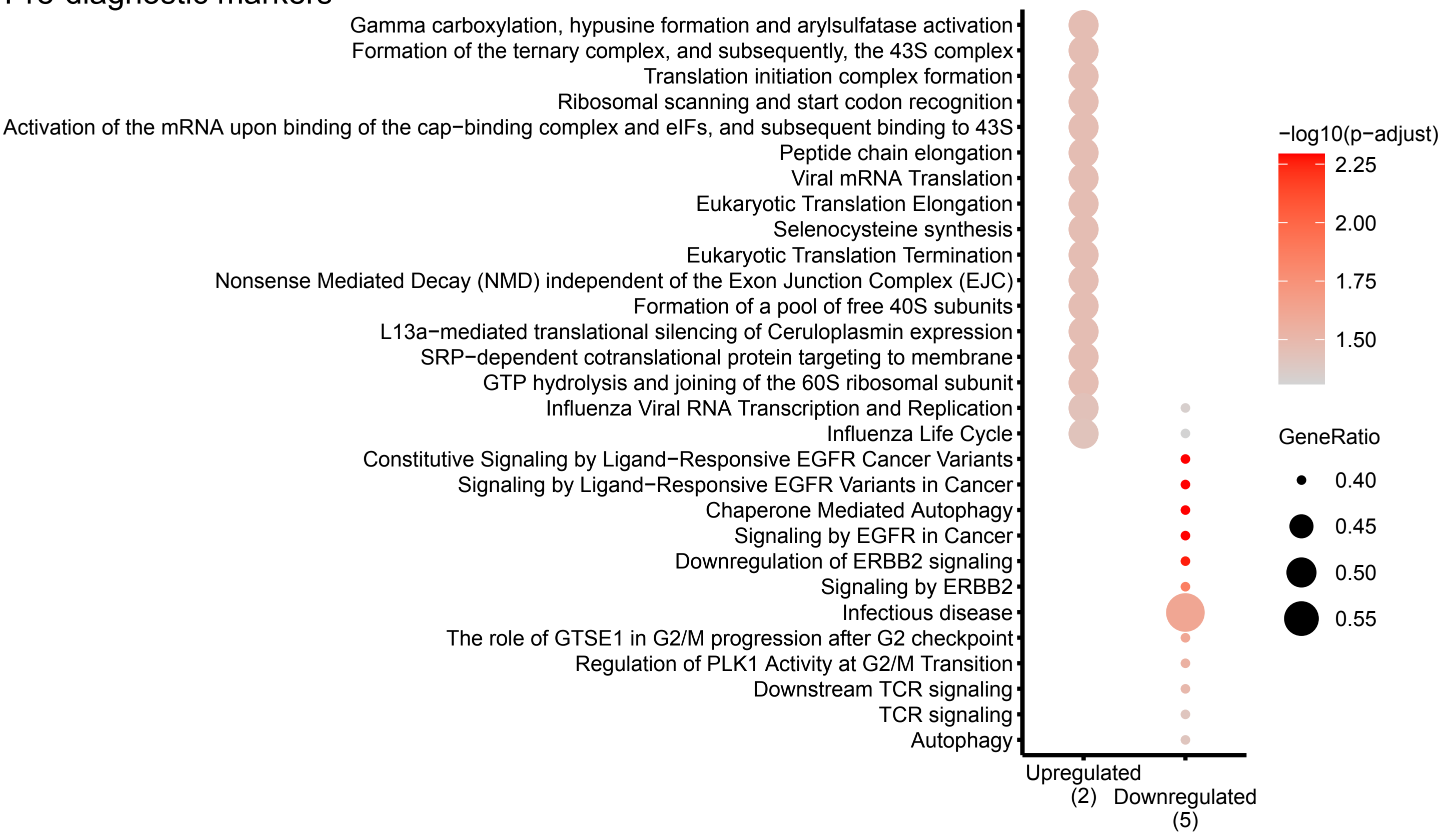

Supplementary figure 6. Pathway enrichment analysis in B cells. Pathways enriched in active CeD and pre-diagnosis markers in B cells identified using the Reactome database (p-value < 0.05). Pathways are on the vertical axis. At the bottom is the direction of expression of the DEGs in B cells. Numbers in brackets indicate the number of DEGs present in all enriched pathways. The size of dot indicates the ratio of the number of genes present in the gene set to the total number of genes used in each pathway.

Active CeD genes

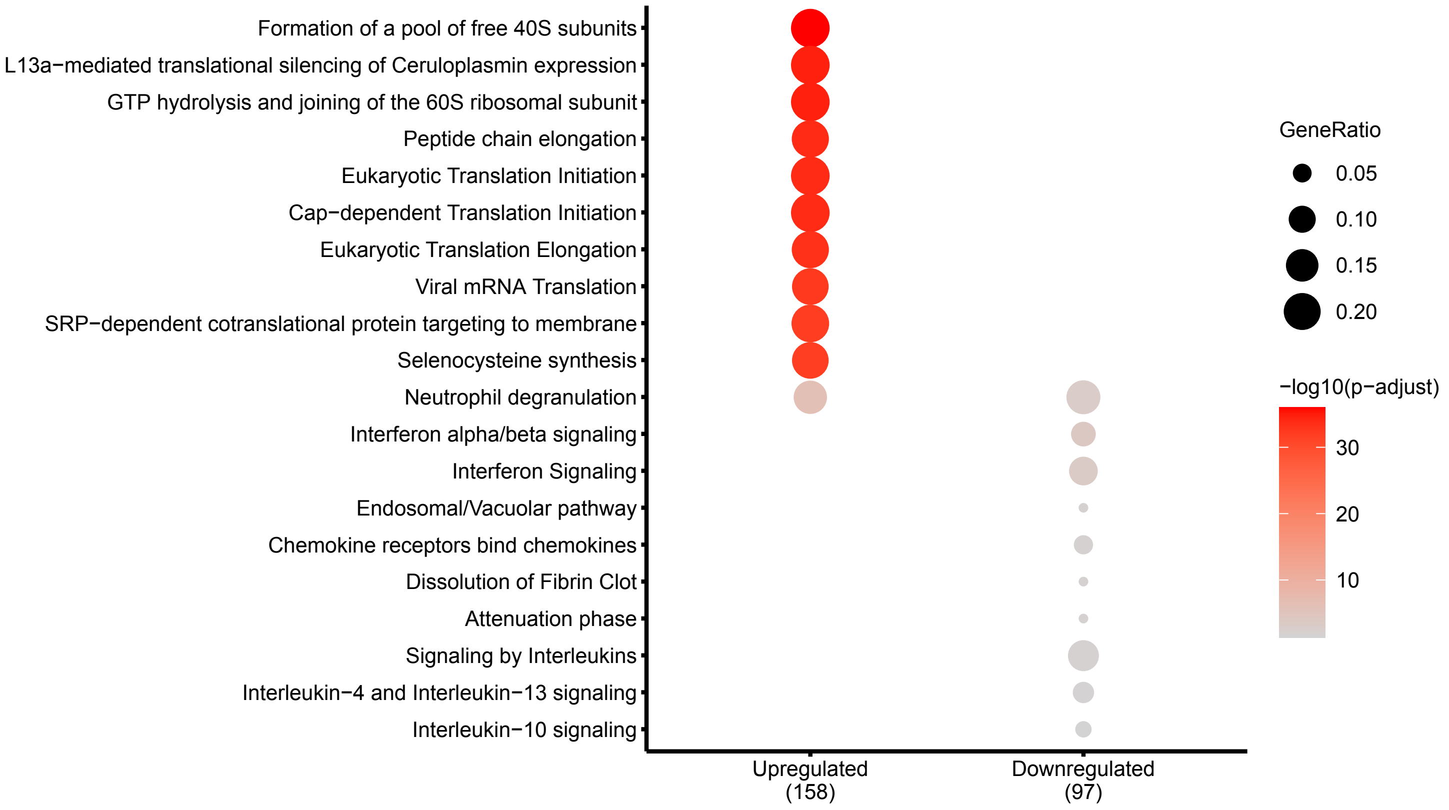

Pre-diagnostic markers

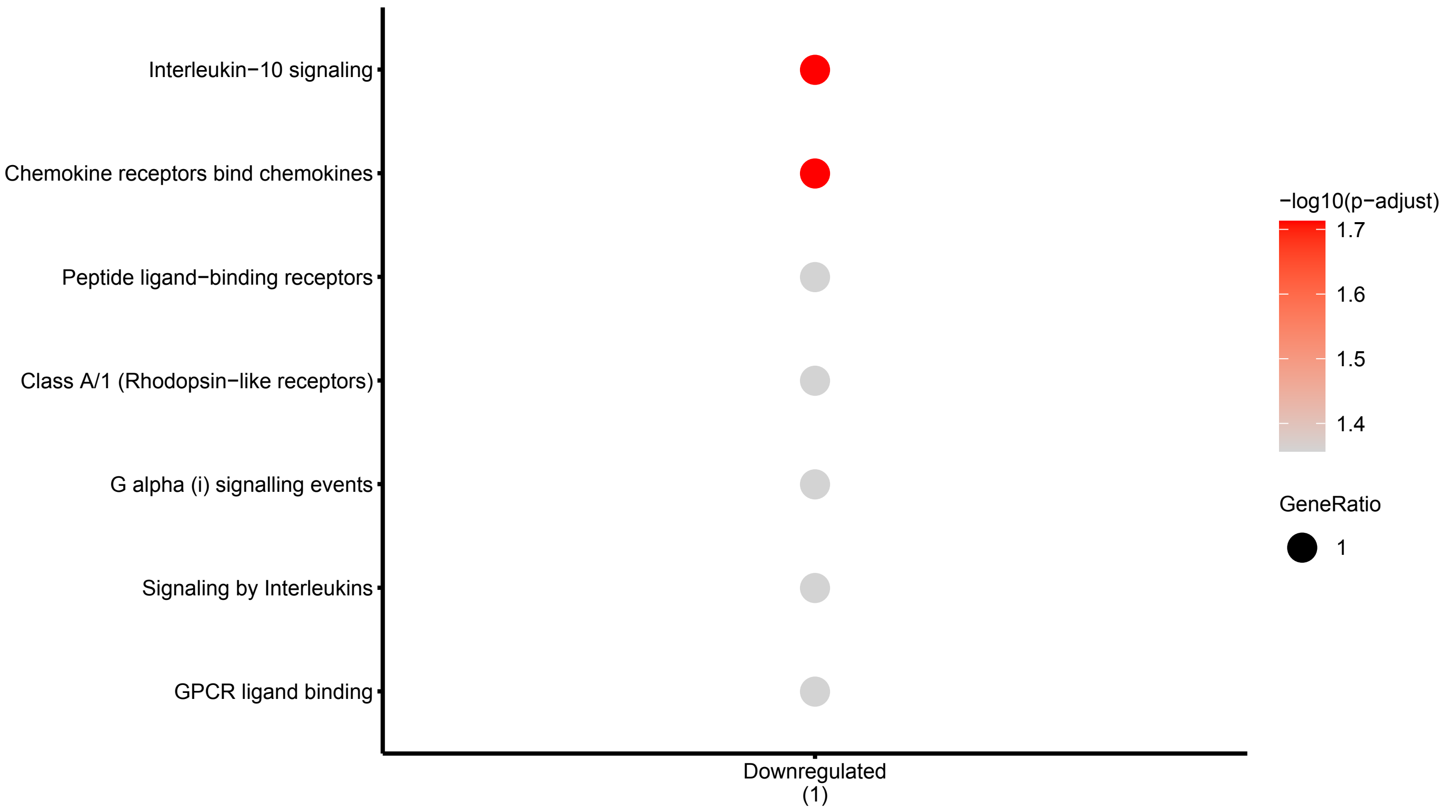

Supplementary figure 7. Pathway enrichment analysis in classical Monocytes. Pathways enriched in active CeD and pre-diagnosis markers in classical Monocytes identified using the Reactome database (p-value < 0.05). Pathways are on the vertical axis. At the bottom is the direction of expression of the DEGs in classical Monocytes. Numbers in brackets indicate the number of DEGs present in all enriched pathways. The size of dot indicates the ratio of the number of genes present in the gene set to the total number of genes used in each pathway.

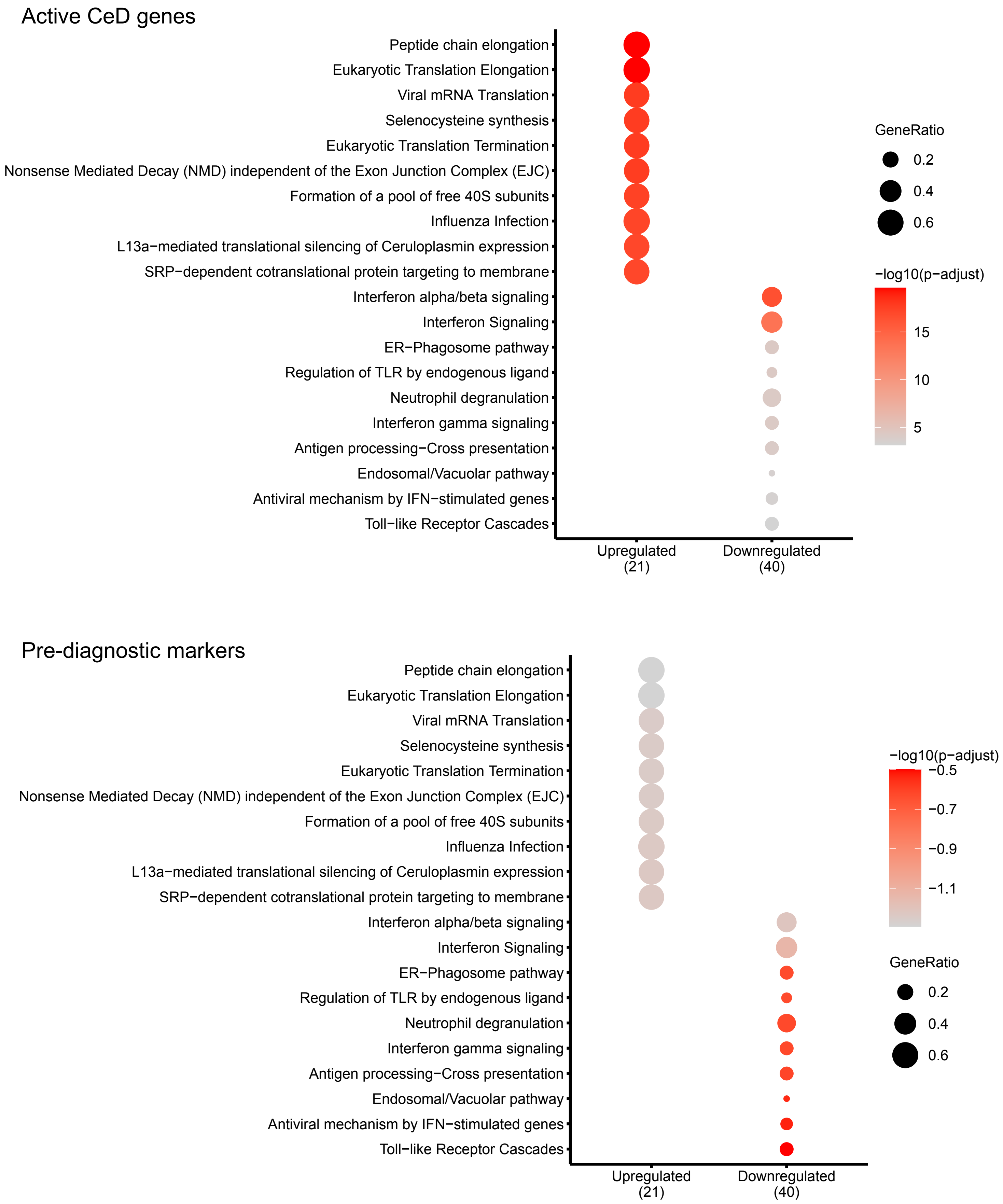

Supplementary figure 8. Pathway enrichment analysis in non-classical Monocytes. Pathways enriched in active CeD and pre-diagnosis markers in non-classical Monocytes identified using the Reactome database (p-value < 0.05). Pathways are on the vertical axis. At the bottom is the direction of expression of the DEGs in non-classical Monocytes. Numbers in brackets indicate the number of DEGs present in all enriched pathways. The size of dot indicates the ratio of the number of genes present in the gene set to the total number of genes used in each pathway.

Active CeD genes

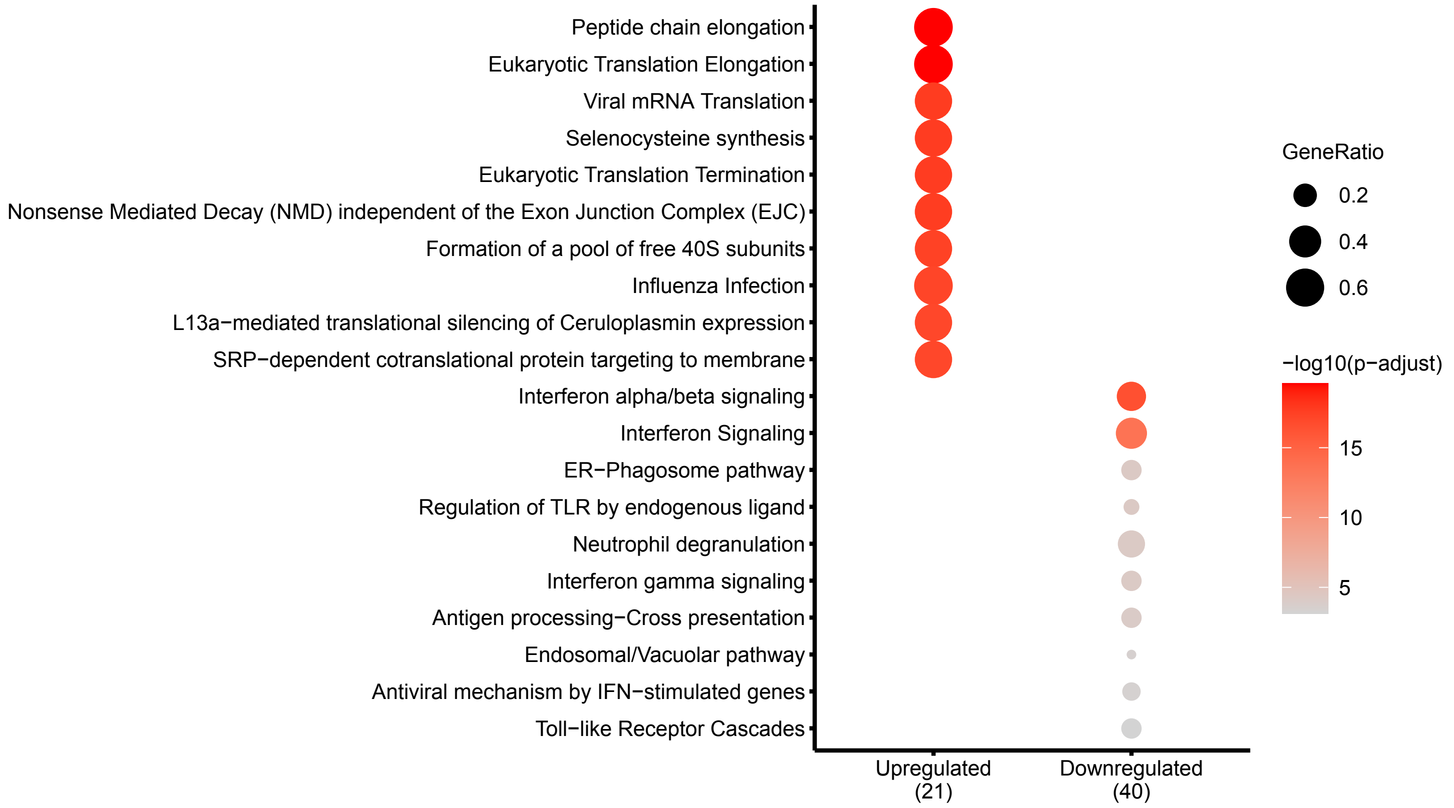

Pre-diagnostic markers

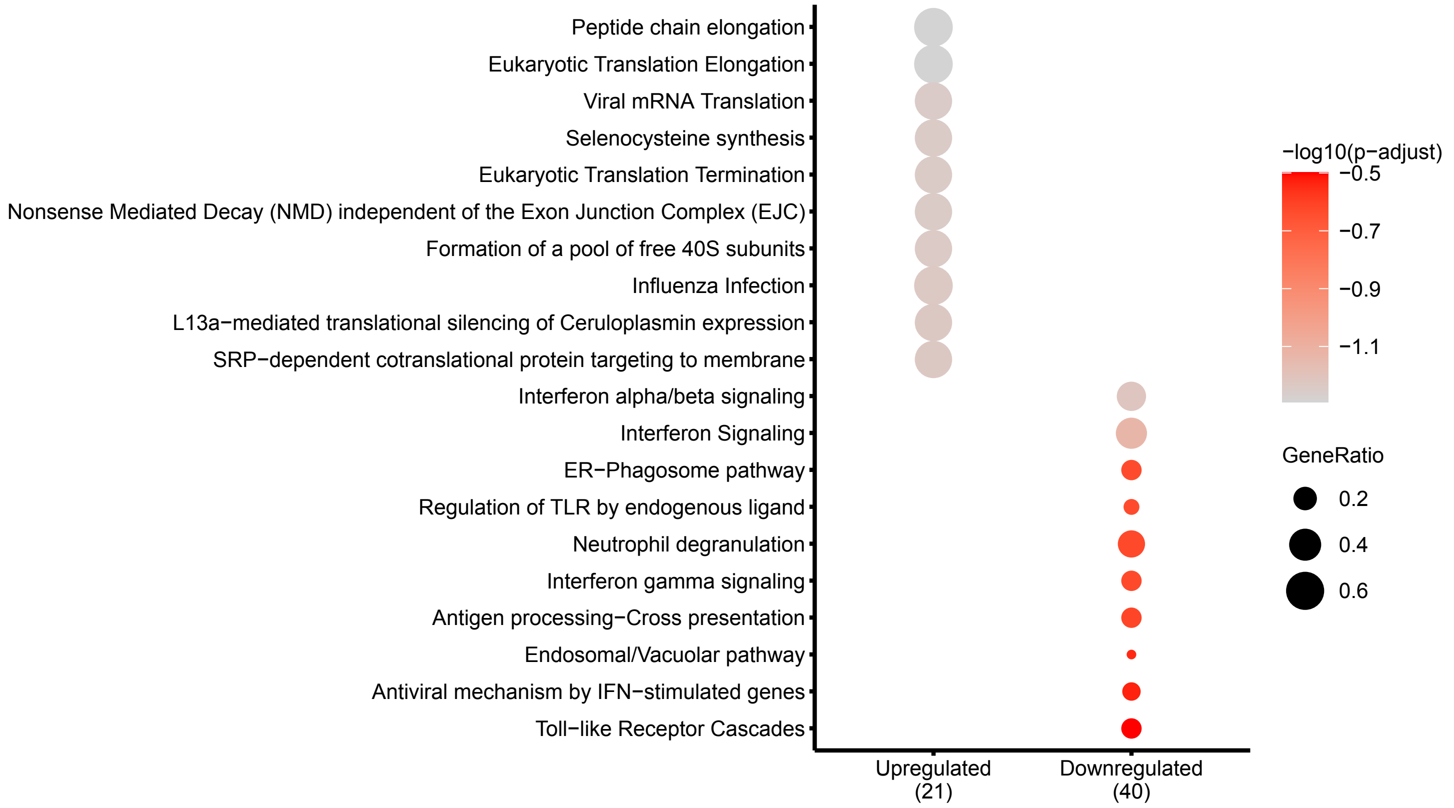

Supplementary figure 9. Pathway enrichment analysis in Dendritic cells. Pathways enriched in active CeD and pre-diagnosis markers in Dendritic cells identified using the Reactome database (p-value < 0.05). Pathways are on the vertical axis. At the bottom is the direction of expression of the DEGs in Dendritic cells. Numbers in brackets indicate the number of DEGs present in all enriched pathways. The size of dot indicates the ratio of the number of genes present in the gene set to the total number of genes used in each pathway.
